# Metabolomic Profiling of Taiwanese Roselle (*Hibiscus sabdariffa*) Leaves and Their Antiproliferative and Insulin Resistance-Ameliorating Potential

**DOI:** 10.3390/foods15101696

**Published:** 2026-05-12

**Authors:** Yu-Hung Liao, Jing-Wen Chen, Yu-Chia Chang, San-Chi Chang, Chun-Han Su

**Affiliations:** 1 Department of Food Science, College of Human Ecology, Fu Jen Catholic University, New Taipei City 242062, Taiwan; 41xd39@gmail.com; 2 Taitung District Agricultural Research and Extension Station, Ministry of Agriculture, Taitung County 950244, Taiwan; jwchen@mail.ttdares.gov.tw; 3 Ph.D. Program of Applied Science and Biotechnology, National Taitung University, Taitung County 950309, Taiwan; 4 Center for Drug Research and Development, College of Human Ecology, Chang Gung University of Science and Technology, Taoyuan City 333324, Taiwan; ycchang03@mail.cgust.edu.tw; 5 Department of Cosmetic Science, Chang Gung University of Science and Technology, Taoyuan City 333324, Taiwan; 6 Department of Microbiology, School of Science, Soochow University, Taipei City 111002, Taiwan; 7 Ph.D. Program in Nutrition and Food Science, College of Human Ecology, Fu Jen Catholic University, New Taipei City 242062, Taiwan

**Keywords:** *Hibiscus sabdariffa*, metabolomics, LC-MS/MS, GC-MS, food by-products, antiproliferative activity, insulin resistance amelioration

## Abstract

Roselle (*Hibiscus sabdariffa*) leaves are the major by-product after harvesting the commercial calyces, and they contain diverse functional metabolites such as phenolic acids and flavonoids. This study aimed to comprehensively characterize the metabolite profiles of the leaves of two Taiwanese roselle varieties (Taitung No. 6 and Taitung No. 3) and to evaluate their antiproliferative and insulin resistance-ameliorating activities. Extracts prepared with 50% EtOH and *n*-hexane were analyzed by untargeted and targeted LC-MS/MS and GC-MS, respectively. Results revealed that the 50% EtOH extracts of both varieties were rich in neochlorogenic acid, cryptochlorogenic acid, rutin, and nicotiflorin. Taitung No. 6 uniquely contained higher levels of anthocyanins including delphinidin-3-*O*-sambubioside and cyanidin-3-*O*-sambubioside. Acidified 50% EtOH extraction increased flavonoid aglycone and polyamine contents. *n*-Hexane extracts were identified as potential sources of vitamin E, α-linolenic acid, squalene, and phytol. Both varieties exhibited antiproliferative activity against BxPC-3 pancreatic cancer cells (IC_50_: 289.2–356.8 μg/mL) and significantly improved the glucose uptake in insulin-resistant HepG2 cells. Neochlorogenic acid and cryptochlorogenic acid were identified as primary active contributors to these bioactivities. This study elucidated the metabolite composition of Taiwanese roselle leaves and provided a scientific basis for the utilization of roselle leaves as a promising functional ingredient.

## 1. Introduction

*Hibiscus sabdariffa* L., commonly known as roselle, sorrel, or *luo shen hua*, is an annual herbaceous subshrub belonging to the family Malvaceae [1]. Roselle is easy to grow in well-drained soils and is widely cultivated in tropical and subtropical regions such as China, Thailand, India, Indonesia, Malaysia, Philippines, Vietnam, Saudi Arabia, Egypt, Sudan, Nigeria, Mexico, and Taiwan [1,2]. In the food industry, calyces (the sepals of a flower) are the most commercially important edible part of roselle for diverse uses, including herbal drinks, beverages, jams, jellies, syrup, confectionery, and flavoring agents [1,2,3]. Due to the presence of anthocyanins, roselle calyces are also used as a natural coloring agent [2]. Although the calyces of roselle possess multiple functions, a large biomass of the roselle plant, such as leaves, remains as by-product after calyx harvesting. From the perspective of sustainable agriculture, the utilization of roselle leaves aligns with the principles of waste valorization by transforming these often-discarded parts of the roselle plant into potential functional ingredients [2,4]. This not only reduces the environmental burden of agricultural waste but also creates potential economic value for the roselle industry.

Roselle leaves are characterized by a mildly tart and green vegetal taste, offering the potential for the development of functional teas [5]. In West African countries such as Niger, Burkina Faso, and Mali, roselle leaves are commonly prepared in sauces and salad. In Niger, fresh roselle leaves can be served with the rice dish as a spice, while dried and pounded old roselle leaves are packaged into small disks for long-term storage [6]. In Sudan, fresh or dried roselle leaves are cooked with onions and peanuts, while in Malaysia, the cooked leaves can be served as a vegetable dish [1]. Besides their culinary uses, roselle leaf infusions are traditionally used as a diuretic, choleretic, febrifuge, or antihypertensive agent in Africa, Mexico, and India [1].

Regarding the chemical composition, roselle leaves contain a range of essential nutrients including vitamins (such as vitamins A, C, and E), minerals (such as calcium and iron), and a relatively high fiber content [2,7,8]. More importantly, the leaves of roselle have been reported to contain diverse bioactive metabolites including phenolic acids, flavonoids, anthocyanins, and organic acids [2,7]. Neochlorogenic acid, cryptochlorogenic acid, and chlorogenic acid were the major phenolic acids detected in the roselle leaves [9,10,11]. For flavonoids, roselle leaves mainly contained quercetin and kaempferol as well as their glycosides such as quercetin-3-rutinoside (rutin), quercetin-3-glucoside, quercetin-3-sambubioside, kaempferol-3-rutinoside, kaempferol-glucoside, and kaempferol-glucoside-rhamnoside [9,10,11,12,13], while other minor flavonoids such as myricetin-3-arabinogalactoside and apigenin-7-glucoside were also found in the leaves of roselle [12,13]. Although anthocyanins were mainly distributed in the calyces of roselle, the leaves were also reported to contain similar anthocyanins as calyces including delphinidin-3-sambubioside, delphinidin-3-glucoside, cyanidin-3-sambubioside, and cyanidin-3-glucoside [9,14]. Organic acids are a group of characteristic metabolites contributing to the sour taste of roselle calyces and leaves, and the leaves were found to contain oxalic acid, citric acid, malic acid, tartaric acid, and ascorbic acid [15].

The presence of various bioactive compounds endows roselle leaf extracts with several bioactivities such as antioxidation, anti-inflammation, antihyperglycemia, antihyperlipidemia, antiproliferation, and so on [2,7,16]. Furthermore, both the aqueous and ethanol extracts of roselle leaves are considered practically non-toxic based on animal studies [17]. The aqueous methanol and ethanol extracts of roselle leaves demonstrated a significant in vitro DPPH and ABTS free radical scavenging activity [10,11,14], while their water and aqueous ethanol extracts also possessed in vitro inhibitory activity on lipid peroxidation [18]. Phenolic acids and flavonoids, especially neochlorogenic acid, chlorogenic acid, cryptochlorogenic acid, rutin, and quercetin-3-glucoside, were considered to be the major antioxidant compounds [10]. Treatments of aqueous methanol extracts of roselle leaves were reported to inhibit the nitric oxide production in lipopolysaccharide-stimulated RAW 264.7 cells and reduce the pro-inflammatory cytokine production in diabetic rats, indicating the anti-inflammatory potential of roselle leaves [11,19]; the same extract was also found to activate the insulin gene expression and enhance insulin production in diabetic rats [19]. Furthermore, treatments with the methanol leaf extract showed a reduction in blood sugar, cholesterol, triglycerides, and LDL cholesterol levels, along with an increase in HDL cholesterol level in diabetic rats [20]; another study using the 80% ethanol extract also exhibited similar antihyperlipidemic effects in rats [18]. The water leaf extract was shown to induce apoptosis of human prostate cancer cells [21], while a polyphenolic-enriched extract of roselle leaves exhibited antiproliferative effects by promoting both apoptosis and autophagy in human melanoma cells [22]. The various health benefits of roselle leaves demonstrate their potential as a functional ingredient, primarily due to their bioactive metabolites particularly phenolic acids and flavonoids [2,7,16].

**Figure 1:**
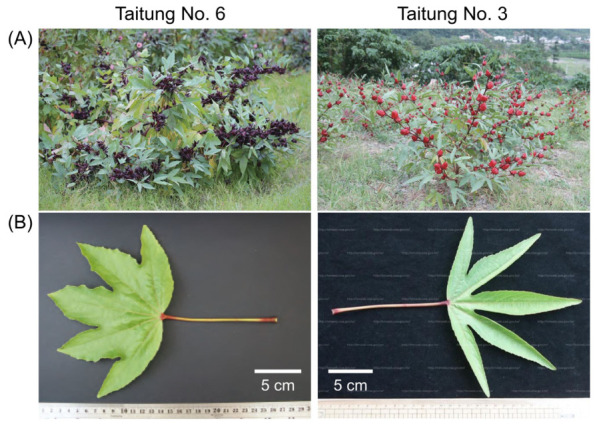
The Taiwanese roselle varieties Taitung No. 6 and Taitung No. 3. (**A**) Field cultivation; (**B**) Leaves [23,24,25].

In Taiwan, roselle is a prominent crop in the region of Taitung, where “Taitung No. 3” serves as a primary traditional roselle variety, and “Taitung No. 6” has emerged as a high-yield roselle variety specifically bred for its rich anthocyanin content (Figure 1) [23]. Despite the extensive research on roselle calyces, the metabolomic diversity and bioactive potential of its leaves (a major by-product after calyx harvesting) remain under-explored. Although some bioactive metabolites in the roselle leaves have been reported, their detailed metabolite composition is still unclear and may vary depending on the variety and origin of plant materials [9,10,11,14]. To address this gap, the present study utilized both LC-MS/MS and GC-MS platforms to perform a holistic and comparative metabolomic characterization of Taitung No. 6 and Taitung No. 3 roselle leaves. Furthermore, their antiproliferative and insulin resistance-ameliorating potentials were evaluated, providing a comprehensive metabolomic profile and evidence for the potential functional applications of roselle leaves.

## 2. Materials and Methods

### 2.1. Reagents and Chemicals

Folin–Ciocalteu’s phenol reagent, 1,1-diphenyl-2-picrylhydrazyl (DPPH), and methylthiazolyldiphenyl-tetrazolium bromide (MTT) were supplied by Sigma-Aldrich (St. Louis, MO, USA). Aluminum chloride was acquired from Alfa Aesar (Heysham, Lancashire, UK). 2-Deoxy-2-[(7-nitro-2,1,3-benzoxadiazol-4-yl)amino]-D-glucose (2-NBDG) was obtained from MedChemExpress (Monmouth Junction, NJ, USA). Metformin hydrochloride was obtained from Macklin (Shanghai, China). Ascorbic acid was purchased from Fisher Chemical (Waltham, MA, USA). Cisplatin was purchased from Acros Organics (Fair Lawn, NJ, USA). Trimethylsilyl (TMS) derivatization reagent (*N*,*O*-bis(trimethylsilyl)trifluoroacetamide with 1% trimethylchlorosilane) was purchased from Macherey-Nagel (Düren, Nordrhein-Westfalen, Germany). Standard compounds including 4-hydroxybenzoic acid, caffeic acid, ferulic acid, gallic acid, *p*-coumaric acid, protocatechuic acid, salicylic acid, shikimic acid, syringic acid, chlorogenic acid, cryptochlorogenic acid, neochlorogenic acid, quercetin, quercetin-3-*O*-glucoside (isoquercitrin), quercetin-3-*O*-rutinoside (rutin), kaempferol, kaempferol-3-*O*-glucoside (astragalin), kaempferol-3-*O*-rutinoside (nicotiflorin), myricetin, cyanidin-3-*O*-glucoside chloride, cyanidin-3-*O*-sambubioside chloride, delphinidin-3-*O*-glucoside chloride, and delphinidin-3-*O*-sambubioside chloride were purchased from Sigma-Aldrich (St. Louis, MO, USA) and Push Biotechnology (Chengdu, China). RPMI 1640 medium, F-12K medium, Dulbecco’s Modified Eagle’s Medium (DMEM), fetal bovine serum (FBS), and antibiotic (penicillin-streptomycin) were obtained from HyClone (South Logan, UT, USA). Recombinant human insulin was acquired from MP Biomedicals (Santa Ana, CA, USA). All additional reagents and chemicals utilized met analytical level of purity.

### 2.2. Materials

The plant materials of two Taiwanese roselle varieties (Taitung No. 6 and Taitung No. 3 of *Hibiscus sabdariffa* (HS)) were cultivated and provided by the Taitung District Agricultural Research and Extension Station of the Ministry of Agriculture (Taitung, Taiwan). The cultivation was conducted following the official Good Agriculture Practice (GAP) guidelines for roselle [26]. The roselle plants were planted in May 2022 and harvested in November 2022 at the mature calyx stage. After harvesting, the leaves were dried by hot air drying in a forced air convection oven at 60 °C for 24 h. The moisture contents of the dried Taitung No. 6 and Taitung No. 3 leaves were 4.8% and 4.3%, respectively. The dried leaves were ground in an electric mill and then sieved through a 50-mesh sieve. The resulting dried leaf powder was stored in a zip-lock bag and protected from light until extraction.

### 2.3. Preparation of Roselle Leaf Extracts

**Figure 2:**
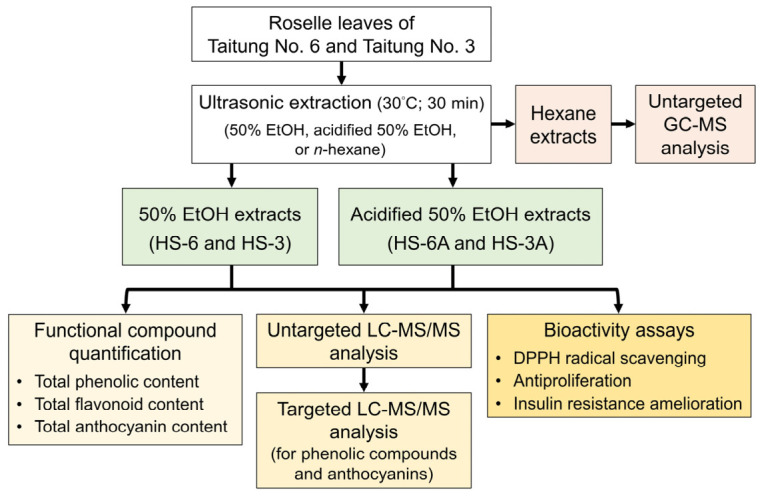
Experimental workflow of sample extraction, metabolomic profiling, and bioactivity evaluation.

Three different solvents including 50% EtOH (ethanol:water, 50:50, *v*/*v*), acidified 50% EtOH (ethanol:water:HCl, 50:49.8:0.2, *v*/*v*/*v*), and *n*-hexane were used as extraction solvents. The 50% EtOH was used to extract high-polar and mid-polar metabolites, and the acidified 50% EtOH was used for comparison because anthocyanins are more stable at an acidic environment [27]. *n*-Hexane was used to extract low-polar and non-polar metabolites. Briefly, five grams of dried leaf powder was mixed with 50 mL of 50% EtOH, acidified 50% EtOH, or *n*-hexane, and then kept in an ultrasonic bath at 30 °C for 30 min. After ultrasonication, the mixtures were filtered, concentrated, and freeze-dried to obtain 50% EtOH extract (named as HS-6 and HS-3 for Taitung No. 6 and Taitung No. 3 roselle, respectively), acidified 50% EtOH extract (named as HS-6A and HS-3A for Taitung No. 6 and Taitung No. 3 roselle, respectively), and hexane extract. These extracts were stored at −20 °C and protected from light for further analysis. The workflow of extraction, metabolomic analysis, and bioactivity evaluation of the leaf extracts is illustrated in Figure 2.

### 2.4. Total Phenolic Content Assay

The total phenolic contents of samples were determined using the Folin–Ciocalteu method [28,29]. The sample (0.5 mL) was mixed with 1 mL of Folin–Ciocalteu’s phenol reagent and 2 mL of Na_2_CO_3_ solution (75 mg/mL, dissolved in water). After 30 min of incubation at room temperature, absorbance measurement at 750 nm was performed. Gallic acid was used as a standard to establish the calibration curve for calculating the total phenolic content of the sample. The total phenolic contents were expressed as milligrams of gallic acid equivalent (GAE) per gram of sample.

### 2.5. Total Flavonoid Content Assay

The total flavonoid contents of samples were determined based on aluminum complex formation [29]. The sample (5 mL) was mixed with 0.3 mL of 5% (*w*/*v*) NaNO_2_ solution, 0.3 mL of 10% (*w*/*v*) AlCl_3_ solution, 4 mL of 4% (*w*/*v*) NaOH solution, and 0.4 mL of water. After 15 min of incubation at room temperature, absorbance measurement at 510 nm was performed. Rutin was used as a standard to establish the calibration curve for calculating the total flavonoid content of the sample. The total flavonoid contents were expressed as milligrams of rutin equivalent (RE) per gram of sample.

### 2.6. Total Anthocyanin Content Assay

The total anthocyanin contents of samples were determined by pH differential spectrophotometry [27]. Briefly, the sample was dissolved in 0.025 M potassium chloride solution (pH 1.0) and 0.4 M sodium acetate buffer (pH 4.5), and the absorbance at 510 nm and 700 nm was measured for each sample solution. The total anthocyanin contents were expressed as milligrams of delphinidin-3-*O*-sambubioside equivalent (D3SE) per gram of sample because D3S is the major anthocyanin in roselle [27]. Calculations were performed using the equation C = ([(A_510_ − A_700_)_pH1.0_ − (A_510_ − A_700_)_pH4.5_] × 597.1)/(23,800 × *l*), where C is the concentration of anthocyanin (mg/mL), 597.1 and 23,800 are the molecular weight (g/mol) and molar extinction coefficient (L/mol·cm) of D3S, respectively, and *l* is the path length (cm).

### 2.7. Untargeted LC-MS/MS Analysis

Untargeted liquid chromatography–tandem mass spectrometry (LC-MS/MS) was performed to analyze the metabolites in the 50% EtOH extracts and acidified 50% EtOH extracts of roselle leaves. Samples were analyzed by an Agilent 1290 Infinity II LC system coupled to an Agilent 6545XT AdvanceBio LC/Q-TOF (quadrupole time-of-flight) mass spectrometer (Agilent Technologies, Santa Clara, CA, USA), equipped with an electrospray ionization (ESI) source. The chromatographic separation was performed with an ACQUITY UPLC BEH Shield RP18 column (2.1 mm × 100 mm, 1.7 μm; Waters, Milford, MA, USA) equipped with a VanGuard pre-column (2.1 mm × 5 mm, 1.7 μm; Waters), and the temperature of column was kept at 40 °C during analysis. The mobile phase comprised water (A) and acetonitrile (B), each acidified with 0.1% (*v*/*v*) formic acid. A constant 0.4 mL/min flow was maintained for elution. The gradient elution was set as follows: 0–1 min, 1–5% B; 1–5 min, 5–10% B; 5–10 min, 10–25% B; 10–15 min, 25–50% B; 15–20 min, 50–99% B; and 20–25 min, 99% B. The column was re-equilibrated at 1% B for 3 min between each run. The samples were dissolved in 50% EtOH at a concentration of 2 mg/mL. Following centrifugation at 13,000× *g* for 10 min, the resulting supernatants were introduced to the LC-MS/MS system. For every run, a 10 μL aliquot was injected. The analyses were performed in positive ion mode and negative ion mode separately for each sample.

MassHunter Workstation software (version B.09.00; Agilent Technologies) was used for LC-MS/MS data acquisition. Data was collected in centroid mode with the MS1 *m*/*z* range of 100–1500, MS2 *m*/*z* range of 50–1500, and a scan rate of 10 spectra/s. Automated iterative data-dependent acquisition (DDA) (three consecutive injections of the same sample) with active exclusion was applied in MS/MS scans, and the top three highest-intensity precursor ions per MS1 scan were selected and fragmented with collision energies of 20 V, 40 V, and 60 V. Metabolite annotation was performed by matching the MS/MS spectral data with open-access online MS/MS spectral libraries including MassBank Europe (MassBank EU), MassBank of North America (MoNA), and Global Natural Products Social Molecular Networking (GNPS). Principal component analysis (PCA) plots and heatmaps were created by using the web-based platform MetaboAnalyst 6.0 [30], with data preprocessing steps including (1) normalization by sum, (2) log transformation (base 10), and (3) auto scaling (mean-centered and divided by the standard deviation of each variable).

### 2.8. Analysis and Quantification of Phenolic Compounds and Anthocyanins

The characteristic phenolic compounds (phenolic acids and flavonoids) and anthocyanins in the 50% EtOH extracts and acidified 50% EtOH extracts of roselle leaves were analyzed by LC-MS/MS according to Piovesana et al. [31] with slight modification. A Shimadzu Nexera X2 UHPLC system interfaced with a Shimadzu LCMS-8045 triple quadrupole mass spectrometer (Shimadzu, Kyoto, Japan) was used with an ESI source running in negative ion mode (for phenolic compound analysis) and positive ion mode (for anthocyanin analysis). The chromatographic separation was carried out with an ACQUITY UPLC BEH Shield RP18 column (2.1 mm × 100 mm, 1.7 μm; Waters, Milford, MA, USA) equipped with a VanGuard pre-column (2.1 mm × 5 mm, 1.7 μm; Waters), and the temperature of the column was kept at 40 °C during analysis. The mobile phase comprised water (A) and acetonitrile (B), each acidified with 0.1% (*v*/*v*) formic acid. A constant 0.4 mL/min flow was maintained for elution. For phenolic compound analysis, the gradient elution condition was set as follows: 0–1 min, 1–5% B; 1–5 min, 5–10% B; 5–10 min, 10–25% B; 10–15 min, 25–50% B; 15–20 min, 50–99% B; and 20–25 min, 99% B. For anthocyanin analysis, the gradient elution condition was set as follows: 0–1 min, 1–5% B; 1–5 min, 5–10% B; 5–10 min, 10–25% B; 10–11 min, 25–99% B; and 11–16 min, 99% B. The column was re-equilibrated at 1% B for 3 min between each run. The samples were dissolved in 50% EtOH. Following centrifugation at 13,000× *g* for 10 min, the resulting supernatants were introduced to the LC-MS/MS system. For every run, a 1 μL aliquot was injected. Identification of the phenolic compounds and anthocyanins was achieved by cross-referencing their retention times and mass spectral data with corresponding authentic standards. For quantification, their contents were determined using the standard curves established based on the peak areas acquired via the multiple reaction monitoring (MRM) mode. The detailed MRM parameters are provided in Tables S1 and S2.

### 2.9. Untargeted GC-MS Analysis

Gas chromatography–mass spectrometry (GC-MS) was used to analyze the metabolites in the hexane extracts of roselle leaves. In brief, the hexane extracts were dissolved in *n*-hexane at a concentration of 2 mg/mL, and trimethylsilyl (TMS) derivatization was performed to obtain TMS-derivatized samples. A Shimadzu Nexis GC-2030 gas chromatograph interfaced with a Shimadzu GCMS-TQ8040 NX triple-quadrupole mass spectrometer (Shimadzu, Kyoto, Japan) was used for sample analysis. The TMS-derivatized sample was centrifuged at 13,000× *g* for 10 min and injected with a volume of 1 μL in split mode (split ratio 1:5). The injector port was maintained at 250 °C, employing helium as the carrier gas at a constant flow of 1 mL/min. Analyte separation was achieved on a Shimadzu SH-I-5Sil MS capillary column (30 m × 0.25 mm i.d., 0.25 μm film thickness), following a programmed oven thermal profile: (a) an initial 1 min kept at 40 °C; (b) a 30 °C/min ramp to 190 °C; (c) a 10 °C/min ramp to 200 °C; (d) a 1 °C/min ramp to 210 °C; (e) a 10 °C/min ramp to 300 °C; and (f) a terminal 15 min hold at 300 °C. The temperature of both the ion source and interface was kept at 280 °C. The ionization was performed via an electron ionization (EI) source at an energy of 70 eV. The monitored *m*/*z* range was 50–800, and the scan rate was 5 spectra/s. NIST 2020 mass spectral library was used for compound identification through mass spectral matching using GCMS Postrun Analysis software (version 4.53SP1; Shimadzu).

### 2.10. DPPH Free Radical Scavenging Activity Assay

The DPPH free radical scavenging activity was used to evaluate the antioxidant potential of samples according to Brand-Williams et al. [32] with slight modification. In brief, 1 mL of DPPH (0.5 mM; dissolved in methanol) was added to 3 mL of the sample (dissolved in methanol). After incubation in the dark at 40 °C for 30 min, the absorbance at 517 nm was measured. Ascorbic acid was used as a positive control. The scavenging activity (%) was calculated using the equation scavenging activity (%) = [1 − (A_sample_/A_control_)] × 100, where A_control_ is the absorbance of the DPPH solution with the baseline control (methanol). EC_50_ values (50% effective concentration; the concentration of samples that scavenge 50% of DPPH free radical) were calculated from dose–effect curves by linear regression.

### 2.11. Antiproliferative Activity Assay

#### 2.11.1. Cell Culture

The human pancreatic cancer cell line BxPC-3 and human lung cancer cell line A549 were obtained from the Bioresource Collection and Research Center (BCRC; Food Industry Research and Development Institute, Hsinchu, Taiwan). RPMI 1640 medium and F-12K medium (both contained 10% FBS and 1% antibiotics) were used as the culture media for BxPC-3 pancreatic cancer cells and A549 lung cancer cells, respectively. The cells were grown at 37 °C in a humidified 5% CO_2_ atmosphere.

#### 2.11.2. Cell Viability Assay

Cells were seeded in a 96-well culture plate (3 × 10^3^ cells/well) and incubated for 24 h before treatment. After the addition of 200 μL of the sample (dissolved in culture medium) and incubation for 48 h, the cell viability was determined by MTT assay to evaluate the antiproliferative activity of the samples. The culture supernatant was removed, and 200 μL of culture medium containing 0.5 mg/mL MTT was added to each well. After incubation at 37 °C for an additional 2 h, the supernatant was removed, and the formazan crystal formed in the cells was dissolved with 100 μL of dimethyl sulfoxide (DMSO). Subsequently, absorbance measurement at 570 nm was performed utilizing a microplate reader (Sunrise; Tecan, Mannedorf, Switzerland). Cisplatin was used as a positive control. Cell viability was normalized to the untreated control group (set as 100%). IC_50_ values (the concentration of samples that inhibit 50% of cell growth) were calculated from dose–effect curves by linear regression.

### 2.12. Insulin Resistance-Ameliorating Assay

The ameliorating effect on insulin resistance was evaluated by establishing an insulin-resistant HepG2 cell model and subsequently determining cellular glucose uptake according to the procedures described by Pavasutti et al. [33] and Wang et al. [34] with slight modification.

#### 2.12.1. Cell Culture

The human HepG2 cell line was obtained from the BCRC of Food Industry Research and Development Institute (Hsinchu, Taiwan). The cells were cultured in the low-glucose DMEM (1 g/L glucose) supplemented with 10% FBS at 37 °C in a humidified 5% CO_2_ atmosphere.

#### 2.12.2. Insulin-Resistant (IR) Cell Model Establishment

Cells were seeded in a 96-well culture plate (4 × 10^4^ cells/well) with low-glucose DMEM (containing 10% FBS) and incubated for 24 h. Subsequently, the medium was replaced with high-glucose DMEM (4.5 g/L glucose) supplemented with 1 μM insulin for 24 h to induce insulin-resistant cells. A control group of normal cells was used for comparison by replacing the medium with insulin-free low-glucose DMEM.

#### 2.12.3. Cellular Glucose Uptake Determination

Insulin-resistant cells were treated with 200 μL of samples (dissolved in low-glucose DMEM) and incubated for 24 h; the cellular glucose uptake was then determined by using 2-NBDG (a fluorescent glucose analog used for monitoring glucose uptake). After removing the medium, the cells were incubated with 100 μL of 100 nM insulin (dissolved in phosphate-buffered saline (PBS)) for 30 min, followed by the addition of 100 μL of 5 μM 2-NBDG and then further incubated for another 1 h. The cells were washed with cold PBS two times, and the fluorescence intensity was then determined using a microplate reader (SpectraMax M2; Molecular Devices, San Jose, CA, USA) with the wavelengths set at 485 nm (excitation) and 528 nm (emission). Metformin was used as a positive control. The percentage of cellular glucose uptake was normalized to the untreated control group of normal cells (set at 100%). Additionally, the viability of the cells treated with different samples was assessed using the MTT assay as described in Section 2.11.2.

### 2.13. Statistical Analysis

All experiments were performed in triplicate using three independent biological replicates (*n* = 3), with each replicate derived from a separate extraction process. Data are presented as mean ± standard deviation (SD). All quantitative data for metabolite contents are expressed on a dry weight basis. Student’s *t*-test was employed to analyze the differences in extraction yields between the two roselle varieties, as well as the total anthocyanin contents between the different extracts within a single variety (Taitung No. 6). For the data of metabolite contents involving multiple factors (variety and extraction solvent), a two-way analysis of variance (ANOVA) was employed to evaluate the main effects of each factor and their interaction. For other comparisons, one-way ANOVA was used. Following ANOVA, Tukey’s honestly significant difference (HSD) post hoc test was performed to address multiple comparisons and identify distinct differences between groups. The assumptions of normality (Shapiro–Wilk test) and homogeneity of variance (Levene’s test) were verified prior to analysis. Differences between groups at *p* < 0.05 were considered as statistically significant.

## 3. Results and Discussion

### 3.1. Extraction Yields

The extraction yields of roselle leaf extracts are summarized in Table 1. The yields of both 50% EtOH extracts (29.67% and 30.81%) and acidified 50% EtOH extracts (33.39% and 32.15%) from both Taitung No. 6 and Taitung No. 3 varieties were significantly higher than those of hexane extracts (5.20% and 5.53%), indicating that the roselle leaves contain a higher proportion of high-polar and mid-polar components than low-polar or non-polar components; this also suggested the potential presence of a high amount of phenolic compounds [35]. A previous study using 50% EtOH as the extraction solvent with different extraction temperatures (60 °C, 80 °C, or 100 °C) reported similar extraction yields ranging from 22% to 32% for roselle leaves [4].

The extraction yield of the acidified 50% EtOH extract of Taitung No. 6 was significantly higher than that of its corresponding 50% EtOH extract, indicating that the mild acidification treatment may enhance extraction efficiency. However, no significant difference in the extraction yield was found between the two roselle varieties. It is important to note that the extraction yield reflects only the total mass of extractable components and does not provide information on the specific chemical composition. Therefore, the quantification of characteristic metabolites, and LC-MS/MS and GC-MS analyses were performed to explore and compare the metabolomic profiles of Taitung No. 6 and Taitung No. 3 roselle leaves.

### 3.2. Total Phenolic, Flavonoid, and Anthocyanin Contents, and DPPH Scavenging Activity

The total amounts of characteristic metabolites of roselle leaves in the 50% EtOH extracts (HS-6 and HS-3 for Taitung No. 6 and Taitung No. 3 roselle, respectively) and acidified 50% EtOH extracts (HS-6A and HS-3A for Taitung No. 6 and Taitung No. 3 roselle, respectively) along with their DPPH free radical scavenging capacity are summarized in Table 2.

The total phenolic contents (TPC) of the extracts ranged from 60.23 to 62.95 mg GAE/g. Although the numerical differences were relatively small, statistical analysis revealed that HS-6 possessed a significantly higher TPC (62.95 mg GAE/g) compared to the acidified extracts HS-6A (60.68 mg GAE/g) and HS-3A (60.23 mg GAE/g), while HS-3 (61.50 mg GAE/g) showed no significant difference from the other extracts. Considering the extraction yields, the TPC of the original dried leaf powder were about 18.68–20.26 mg GAE/g, which were similar to the TPC (18.59–22.49 mg GAE/g) of the leaves from nine roselle accessions cultivated in Guam, USA [14].

For the total flavonoid contents (TFC), significant differences were observed between the two roselle varieties and extraction conditions. Taitung No. 3 extracts (HS-3 and HS-3A) exhibited significantly higher TFC compared to their corresponding Taitung No. 6 counterparts. HS-3 contained the highest TFC (101.03 mg RE/g), followed by HS-6 (90.76 mg RE/g) and HS-3A (88.01 mg RE/g), while the lowest TFC was found in HS-6A (78.60 mg RE/g). These TFC values were higher than those reported for the roselle leaf extracts (6–77 mg/g) obtained by supercritical fluid extraction at 40 °C [4]. It was noted that the acidified 50% EtOH extracts (HS-6A and HS-3A) contained significantly lower TFC than their corresponding 50% EtOH extracts (HS-6 and HS-3); this outcome may be attributed to the changes in the solubility and acid hydrolysis ratio of flavonoids in the presence of acid in the extraction solvent. The method used for TFC determination was based on aluminum complex formation, and therefore the quantification results may also be affected by the structural diversity of flavonoids and their structure changes during acidic extraction treatment [36]. In addition, the TFC was about 1.3–1.6 fold of the TPC in the roselle leaves; this finding is consistent with previous studies indicating that the TFC was higher than the TPC in the roselle leaves [21,37], suggesting that the leaves of roselle could be a rich source of flavonoids.

The total anthocyanin contents (TAC) were only detectable in HS-6 (1.23 mg D3SE/g) and HS-6A (1.34 mg D3SE/g). This is reasonable because the variety Taitung No. 6 is known for its high anthocyanin contents in its calyces (30.6 mg/g dry weight) compared to that in Taitung No. 3 calyces (4.0 mg/g dry weight) [23]. The TAC level of the Taitung No. 6 leaves was similar to those of roselle accessions containing relatively higher amounts of TAC [14]. Moreover, although anthocyanins are known to be more stable in an acidic environment [27], the addition of acid in the 50% EtOH extraction solvent did not significantly increase the TAC; this observation can be attributed to the inherent acidity of roselle leaves (pH about 2.6 in the 50% EtOH solvent), which is close to the pH (about 2.2) of using acidified 50% EtOH extraction of the roselle leaves.

The DPPH free radical scavenging activity of the roselle leaf extracts was relatively similar to each other (with EC_50_ values ranging from 165.3 to 198.3 μg/mL), but the EC_50_ value of the acidified 50% EtOH extract of Taitung No. 3 (HS-3A) was significantly higher than those of the other extracts, indicating a lower free radical scavenging potency. The EC_50_ value of ascorbic acid (positive control) was 7.37 ± 0.91 μg/mL. Although the DPPH scavenging activity of these roselle leaf extracts was much weaker than that of ascorbic acid, their DPPH scavenging potency was similar to those of other roselle varieties [14]. A previous study indicated that the roselle leaves possessed similar DPPH scavenging capacity as another 24 common leafy vegetables [37], demonstrating the potential use of roselle leaves as a source of antioxidants. In this study, the DPPH scavenging activity of Taitung No. 6 appeared to be stronger than Taitung No. 3, indicating that the presence of anthocyanins could contribute to the antioxidant activity by donating hydrogen atoms and electrons to neutralize free radicals [16,38]. However, no direct relationship was found between the total contents of the major functional compounds (TPC and TFC) and the DPPH free radical scavenging activity of these leaf extracts.

### 3.3. Untargeted LC-MS/MS Analysis of the Metabolites in the 50% EtOH Extracts and Acidified 50% EtOH Extracts

Both positive ion and negative ion modes of untargeted LC-MS/MS analysis were conducted to characterize the metabolomic profiles and differences in the 50% EtOH extracts and acidified 50% EtOH extracts of Taitung No. 6 and Taitung No. 3 roselle leaves. The approach of using dual ionization modes was essential to increase the metabolite coverage and ensure the comprehensive detection of chemically diverse constituents; the positive ion mode was crucial for detecting basic or cationic species such as anthocyanins and nitrogen-containing compounds, while the negative ion mode provided superior sensitivity for acidic compounds such as phenolic acids and organic acids.

**Figure 3:**
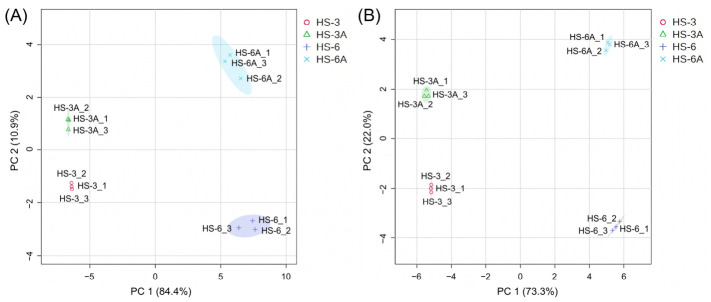
Principal component analysis (PCA) score plots of the metabolomes of the 50% EtOH extracts (HS-6 and HS-3) and acidified 50% EtOH extracts (HS-6A and HS-3A) of the leaves from Taitung No. 6 and Taitung No. 3 roselle. (**A**) Positive ion mode data; (**B**) Negative ion mode data.

The principal component analysis (PCA) score plots are shown in Figure 3. In the positive ion mode, the first principal component (PC1) and second principal component (PC2) explained 84.4% and 10.9% of the variance, respectively. In the negative ion mode, the PC1 and PC2 explained 73.3% and 22.0% of the total metabolomic variance, respectively. Clear and consistent clustering was observed in both modes. HS-6 and HS-3 samples exhibited wide separation along the PC1 axis, indicating that the genetic background is the primary factor determining the metabolomic variation. The acidified 50% EtOH extracts (HS-6A and HS-3A) were clearly distinguished from their respective non-acidified counterparts (HS-6 and HS-3) along the PC2 axis. This suggested that the acidification induced consistent changes in the leaf metabolite profiles, with similar trends observed across both varieties.

**Figure 4:**
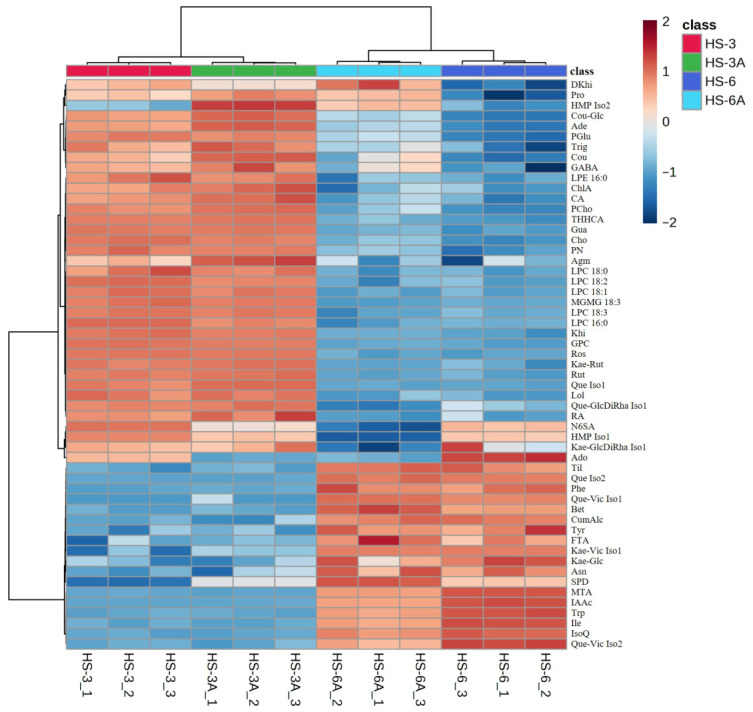
Heatmap of the metabolomes of the 50% EtOH extracts (HS-6 and HS-3) and acidified 50% EtOH extracts (HS-6A and HS-3A) of the leaves from Taitung No. 6 and Taitung No. 3 roselle in positive ion mode. Each row in the heatmap represents a metabolite, and each column represents a sample. The color scale from blue to red indicates relative abundance (relative peak area) from low to high. The original relative peak areas are summarized in Table S3, and the abbreviations for the original compound names are listed in Table S4.

**Figure 5:**
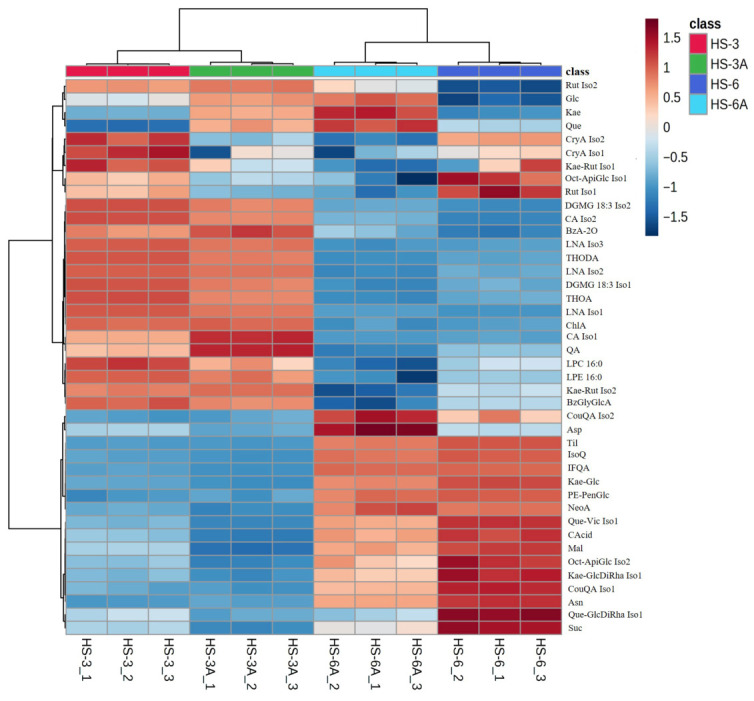
Heatmap of the metabolomes of the 50% EtOH extracts (HS-6 and HS-3) and acidified 50% EtOH extracts (HS-6A and HS-3A) of the leaves from Taitung No. 6 and Taitung No. 3 roselle in negative ion mode. Each row in the heatmap represents a metabolite, and each column represents a sample. The color scale from blue to red indicates relative abundance (relative peak area) from low to high. The original relative peak areas are summarized in Table S5, and the abbreviations for the original compound names are listed in Table S6.

Diverse metabolites, including amino acids and their derivatives, organic acids, phenolic acids, flavonoids and their glycosides, sugars, cholines and betaines, triterpenoid derivatives, and various lipids (phospholipids and glycolipids), were detected by untargeted LC-MS/MS. The heatmaps (Figure 4 and Figure 5) revealed the relative abundance characteristics of metabolites in the roselle leaf extracts and highlighted the differences resulting from variety and acidified extraction.

In the heatmap of positive ion mode (Figure 4), the HS-6 group samples exhibited apparently higher accumulation of various amino acids and amino acid derivatives compared to the HS-3 group, such as phenylalanine (Phe), isoleucine (Ile), tryptophan (Trp), tyrosine (Tyr), asparagine (Asn), spermidine (SPD), and betaine (Bet). Conversely, the HS-3 group samples contained higher relative amounts of phospholipids (LPC 16:0, LPC 18:3, LPC 18:2, LPC 18:1, LPC 18:0, and LPE 16:0), amino acid derivatives (such as choline (Cho), agmatine (Agm), γ-aminobutyric acid (GABA)), and khivorin (Khi; a triterpenoid derivative). Interestingly, both HS-3 and HS-3A possessed higher relative contents of certain phenolic acids (such as rosmarinic acid (RA), chlorogenic acid (ChlA), and caffeic acid (CA)) and certain flavonoid diglycosides (such as rutin (Rut) and kaempferol-3-*O*-rutinoside (Kae-Rut)) than those of HS-6 and HS-6A. These observations suggested the metabolic differences between Taitung No. 6 and Taitung No. 3 varieties, where Taitung No. 6 was characterized by a richer amino acid metabolic profile, while Taitung No. 3 showed an enrichment of phospholipids and specific phenolic acids and flavonoid glycosides.

In the heatmap of negative ion mode (Figure 5), higher relative contents of some phenolic acids and organic acids were found in the HS-6 group samples, such as neochlorogenic acid (NeoA), malic acid (Mal), and citric acid (CAcid). The HS-6 group samples also accumulated higher relative amounts of some flavonoid glycosides including isoquercitrin (IsoQ), kaempferol-7-*O*-glucoside (Kae-Glc), and tiliroside (Til). Similar to the results of positive ion mode data, the HS-3 group samples contained higher relative contents of certain types of flavonoid diglycosides such as rutin or isomer (Rut Iso2) and kaempferol-3-*O*-rutinoside or isomer (Kae-Rut Iso2), as well as chlorogenic acid (ChlA), phospholipids (LPC 16:0 and LPE 16:0), and glycolipids (DGMG 18:3 Iso1 and DGMG 18:3 Iso2). These findings revealed varietal feature patterns: Taitung No. 6 tended to accumulate neochlorogenic acid and some specific organic acids, whereas Taitung No. 3 may favor the accumulation of chlorogenic acid, and phospholipid and glycolipid metabolites. Furthermore, different profiles of flavonoid glycosides were also noted between the Taitung No. 6 and Taitung No. 3 leaves.

Next, the metabolomic differences between the 50% EtOH extracts and acidified 50% EtOH extracts were compared to evaluate the effects of acidified extraction on the metabolite composition of extracts. In the heatmap of positive ion mode, the acidified 50% EtOH extracts (HS-6A and HS-3A) exhibited a markedly higher accumulation of the polyamine spermidine (SPD) compared to their non-acidified counterparts. In contrast, the 50% EtOH extracts (HS-6 and HS-3) retained higher relative levels of tryptophan (Trp), adenosine (Ado), and 5-*S*-methylthioadenosine (MTA). This suggested that the acidified extraction solvent may enhance the recovery of certain polyamines while potentially promoting the degradation or transformation of particular nucleosides and amino acids.

The heatmap of negative ion mode data revealed that when compared to the 50% EtOH extracts, the acidified 50% EtOH extracts possessed obviously higher relative amounts of kaempferol (Kae) and quercetin (Que), along with elevated levels of glucose (Glc). In contrast, the 50% EtOH extracts possessed higher relative contents of sucrose (Suc), malic acid (Mal), citric acid (CAcid), and certain flavonoid glycosides including kaempferol-7-*O*-glucoside (Kae-Glc), Kae-GlcDiRha Iso1, and several quercetin glycosides (such as Rut Iso1, Que-Vic Iso1, and Que-GlcDiRha Iso1) than those in the acidified 50% EtOH extracts. These findings indicated that the acidified extraction greatly enhanced the release of flavonoid aglycones, especially kaempferol and quercetin, and these trends may result from the hydrolysis of flavonoid glycosides in the acidified environment [39], which simultaneously triggered the apparent hydrolysis of sucrose into glucose.

### 3.4. Contents of the Characteristic Phenolic Compounds in the 50% EtOH Extracts and Acidified 50% EtOH Extracts

Based on the results of untargeted LC-MS/MS analyses along with common functional compounds found in the leaves of roselle in previous studies [9,10,11,12,13,14], four anthocyanins, twelve phenolic acids, and seven flavonoids were selected for quantitative analysis by LC-triple quadrupole, and the results are summarized in Table 3. The results of anthocyanin quantification showed that the 50% EtOH extract and the acidified 50% EtOH extract of Taitung No. 6 leaves contained 0.900 and 0.981 mg/g delphinidin-3-*O*-sambubioside (D3S) and 0.289 and 0.320 mg/g cyanidin-3-*O*-sambubioside (C3S), respectively. Accounting for the extraction yield of the acidified 50% EtOH extract, the D3S and C3S contents in the original Taitung No. 6 dried leaf powder were 0.328 mg/g and 0.107 mg/g, respectively. The D3S content was notably higher, while the C3S content fell within the reported ranges (0.01–0.24 mg/g for D3S and 0.01–0.61 mg/g for C3S) of the leaves from various roselle accessions cultivated in Guam, USA [14]. Furthermore, two minor anthocyanins (<0.1 mg/g) including delphinidin-3-*O*-glucoside (D3G) and cyanidin-3-*O*-glucoside (C3G) were also detected in the leaf extracts of Taitung No. 6. These findings indicated that the major anthocyanin composition of the Taitung No. 6 leaves is similar to that of roselle calyces [35].

The combined contents of those four anthocyanins in the Taitung No. 6 leaf extracts were similar to the total anthocyanin contents (TAC) as shown in Table 2. Although the TAC of Taitung No. 3 leaf extracts was not detectable by the traditional spectrophotometry method, minor amounts (<0.1 mg/g) of D3S and C3S, as well as trace amounts of D3G, were detected in the Taitung No. 3 leaf extracts by the LC-MS/MS method. Generally, there was no significant difference in the contents of different anthocyanins between the 50% EtOH extracts (HS-6 and HS-3) and the corresponding acidified 50% EtOH extracts (HS-6A and HS-3A), further confirming that the enhancing effects on the anthocyanin content by the acidified extraction condition used in this study is limited.

Among the twelve quantified phenolic acids, chlorogenic acid isomers were the predominant ones, especially neochlorogenic acid. The contents of neochlorogenic acid, cryptochlorogenic acid, and chlorogenic acid in HS-6 were 6.461, 3.067, and 0.242 mg/g, respectively, while their contents in HS-3 were 5.637, 3.378, and 0.345 mg/g, respectively. These findings were consistent with previous studies, suggesting that these three chlorogenic acid isomers were the major phenolic acids in the leaves of roselle [9,10,11]. Furthermore, the observed levels were comparable to the reported ranges for roselle leaves from various worldwide varieties (2.87–7.63 mg/g for neochlorogenic acid, 1.14–2.72 mg/g for cryptochlorogenic acid, and 0.35–1.49 mg/g for chlorogenic acid in 70% MeOH extracts) [10,11]. For Taitung No. 6 and Taitung No. 3, a varietal difference was found in the contents of the chlorogenic acid isomers; HS-6 contained significantly higher amounts of neochlorogenic acid but significantly lower amounts of cryptochlorogenic acid and chlorogenic acid than those of HS-3. Moreover, the contents of these chlorogenic acid isomers were lower in the acidified 50% EtOH extracts (HS-6A and HS-3A) compared to the corresponding 50% EtOH extracts (HS-6 and HS-3); the reason could be due to the lower pH during extraction that may promote the transformation of chlorogenic acids into their corresponding derivatives [40].

In addition to the chlorogenic acid compounds, syringic acid and shikimic acid were found as the second major phenolic acids in both Taitung No. 6 and Taitung No. 3 leaf extracts. The contents of syringic acid in HS-6, HS-6A, HS-3, and HS-3A were 0.047, 0.063, 0.062, and 0.069 mg/g, respectively, while the contents of shikimic acid in these four extracts were 0.039, 0.037, 0.031, and 0.031 mg/g, respectively. In addition, the leaf extracts of Taitung No. 3 contained significantly higher amounts of caffeic acid (0.079 mg/g for HS-3 and 0.083 mg/g for HS-3A) and protocatechuic acid (0.031 mg/g for HS-3 and 0.027 mg/g for HS-3A) than those of Taitung No. 6. Furthermore, five other minor phenolic acids (≤0.02 mg/g) were also found in both Taitung No. 6 and Taitung No. 3 leaf extracts, including gallic acid, 4-hydroxybenzoic acid, *p*-coumaric acid, ferulic acid, and salicylic acid. Generally, for most minor phenolic acids, there was no significant difference in their contents between the 50% EtOH extracts (HS-6 and HS-3) and the corresponding acidified 50% EtOH extracts (HS-6A and HS-3A), indicating that the impact of acidified extraction condition on the stability of these minor compounds was relatively weak.

For the flavonoids, quercetin, kaempferol, and myricetin, as well as several glycosides of quercetin and kaempferol were determined. Based on the results, rutin (quercetin-3-*O*-rutinoside) accounted for the highest proportion among different flavonoid glycosides, and its contents were significantly higher in Taitung No. 3 (12.819 mg/g for HS-3 and 10.893 mg/g for HS-3A) than in Taitung No. 6 (9.894 mg/g for HS-6 and 8.899 mg/g for HS-6A). The second rich flavonoid glycosides were nicotiflorin (kaempferol-3-*O*-rutinoside) and isoquercitrin (quercetin-3-*O*-glucoside), and the leaf extracts of Taitung No. 6 contained significantly higher amounts of isoquercitrin (2.879 mg/g for HS-6 and 2.211 mg/g for HS-6A) but significantly lower amounts of nicotiflorin (2.873 mg/g for HS-6 and 2.675 mg/g for HS-6A) than those of Taitung No. 3. Another glycoside of kaempferol, astragalin (kaempferol-3-*O*-glucoside), was relatively minor in both extracts of Taitung No. 6 and Taitung No. 3 (0.493 mg/g for HS-6 and 0.259 mg/g for HS-3). Furthermore, the observed levels of major flavonoid glycosides were comparable to those reported for roselle leaves from several worldwide varieties (7.2–20.5 mg/g for rutin, and 0.97–4.70 mg/g for isoquercitrin in 70% MeOH extracts) [10].

Among the flavonoid aglycones, the content of quercetin was relatively higher than the others, and its contents in HS-6 and HS-6A were 0.259 mg/g and 0.443 mg/g, respectively, while in HS-3 and HS-3A were 0.235 mg/g and 0.300 mg/g, respectively. Minor amounts of kaempferol were detected in the leaf extracts of Taitung No. 6 (0.062 mg/g for HS-6 and 0.109 mg/g for HS-6A) and Taitung No. 3 (0.066 mg/g for HS-3 and 0.085 mg/g for HS-3A), while the contents of myricetin were much lower than quercetin and kaempferol in both roselle varieties. Comparing the contents of flavonoid glycosides and aglycones, HS-6 and HS-3 had higher contents of flavonoid glycosides but lower amounts of aglycones than their corresponding acidified counterparts (HS-6A and HS-3A), and these findings appeared to be reasonable because flavonoid glycosides tend to be hydrolyzed into aglycones under an acidic environment [39].

The differences in the phenolic compound contents such as anthocyanins and some phenolic acids and flavonoids between Taitung No. 6 and Taitung No. 3 may result from their different genotypes [9,10,11,14], and the results proved that the leaves of Taitung No. 6 were relatively rich in anthocyanins especially D3S and C3S. The major anthocyanins (D3S and C3S), phenolic acids (neochlorogenic acid, cryptochlorogenic acid, and chlorogenic acid) and flavonoids (quercetin glycosides and kaempferol glycosides) in the leaves of Taitung No. 6 and Taitung No. 3 have been reported to possess several bioactivities such as antioxidant, anti-inflammatory, antiproliferative, and antihyperglycemic activities [2,7,16]; these observations suggested that the bioactivities of 50% EtOH extracts of both Taitung No. 6 and Taitung No. 3 leaves deserve further investigations.

### 3.5. Untargeted GC-MS Analysis of the Metabolites in the Hexane Extracts

Hexane extracts were prepared for the analysis of low-polar and non-polar metabolites in the leaves of Taitung No. 6 and Taitung No. 3 roselle. A total of 37 characteristic metabolites were tentatively identified by GC-MS analysis (Table 4), including thirteen fatty acids, six terpenoids, three steroids, six alkanes, and others such as alcohols and organic acids. The relative abundances of these compounds were compared based on the ratio of their respective chromatographic peak areas.

The fatty acids in the hexane extracts of the leaves of Taitung No. 6 and Taitung No. 3 roselle were mainly composed of palmitic acid (C16:0), α-linolenic acid (C18:3), linoleic acid (C18:2), and stearic acid (C18:0); the percentages of peak area (potential relative content) of these four fatty acids were 13.42%, 12.59%, 3.44%, and 2.51% for Taitung No. 6, and 15.92%, 11.44%, 3.14%, and 3.93% for Taitung No. 3, respectively. In addition, nine other minor fatty acids (each < 1%), including oleic acid (C18:1) and eight saturated fatty acids with different carbon chain lengths (C14:0, C17:0, C20:0, C22:0, C23:0, C24:0, C25:0, and C26:0), were also detected in both Taitung No. 6 and Taitung No. 3 roselle leaves.

Six terpenoids were found in the hexane extracts of Taitung No. 6 and Taitung No. 3 leaves. Squalene (an acyclic triterpene) and phytol (an acyclic diterpene alcohol) were the predominant terpenoids, and Taitung No. 6 contained a relatively higher proportion of squalene (8.34%) and phytol (8.23%) than those of Taitung No. 3 (4.90% squalene and 3.59% phytol). Other minor terpenoids (each < 2%) included three pentacyclic triterpenoids (α-amyrin, lupeol, and 24-norursa-3,12-diene) and one acyclic diterpene (neophytadiene). Furthermore, the three steroids detected in the hexane extracts of Taitung No. 6 and Taitung No. 3 roselle leaves included β-sitosterol, stigmasterol, and campesterol, with the peak area ratios of 3.83%, 2.75%, and 1.10% for Taitung No. 6, and 3.70%, 2.48%, and 1.09% for Taitung No. 3, respectively.

Six long-chain alkanes, including tetracosane (C24), nonacosane (C29), dotriacontane (C32), hexatriacontane (C36), tetratetracontane (C44), and tetrapentacontane (C54), were detected as the main alkane compounds in the leaf hexane extracts of Taitung No. 6 and Taitung No. 3, which may be derived from the cuticular waxes of the leaves. The peak area ratios of tetracosane, nonacosane, dotriacontane, hexatriacontane, tetratetracontane, and tetrapentacontane were 0.19%, 1.04%, 0.27%, 4.20%, 0.77%, and 2.39% for Taitung No. 6, and 0.12%, 0.67%, 0.25%, 4.85%, 0.99%, and 3.65% for Taitung No. 3, respectively. Regarding other types of compounds, the common form of vitamin E (α-tocopherol) accounted for a relatively higher ratio in the leaf hexane extracts of both Taitung No. 6 (18.13%) and Taitung No. 3 (23.22%). This finding was consistent with a previous study indicating that the leaves of roselle were rich in α-tocopherol [41]. Moreover, other detected compounds included γ-tocopherol, alcohols (7-methoxy-3,7-dimethyloctan-1-ol and *n*-heptadecan-1,2-diol), organic acids (lactic acid and tartronic acid), a fatty acid amide (*cis*-11-eicosenamide), a pyranone (3-hydroxy-2,3-dihydromaltol), and a glyceride (1-linolenoylglycerol).

Overall, based on the relative chromatographic peak area ratios, the most abundant functional metabolites in the hexane extracts of Taitung No. 6 and Taitung No. 3 roselle leaves included two essential fatty acids (α-linolenic acid and linoleic acid), vitamin E, two terpenoids (squalene and phytol), and two phytosterols (β-sitosterol and stigmasterol). α-Linolenic acid is an essential omega-3 polyunsaturated fatty acid and a fundamental structural component of cell membranes, where it regulates membrane fluidity, permeability, and receptor activation [42]. Furthermore, α-linolenic acid has been shown to modulate the synthesis of hormones that regulate inflammation and vascular contraction; α-linolenic acid has also been reported to exhibit anti-inflammatory, cardioprotective, and anticancer activities, contributing to the prevention of heart disease, stroke, and various malignancies [42]. Vitamin E, particularly its most active form α-tocopherol, is a potent lipophilic antioxidant that preserves cell membrane integrity by scavenging reactive oxygen species and interrupting lipid peroxidation chains [43]; vitamin E has also been reported to exert systemic health benefits through immune modulation and the inhibition of pro-inflammatory cytokines, thereby reducing the risks of cardiovascular and neurodegenerative diseases [43]. Squalene is a critical metabolic intermediate in sterol biosynthesis, and it may serve as a powerful antioxidant and anti-inflammatory agent, protecting tissues from oxidative stress and providing photoprotection against UV-induced damage [44]. Phytol is derived from chlorophyll, possessing a broad spectrum of bioactivities, such as anti-inflammatory, antioxidant, and antimicrobial properties [45]. Phytol has been reported to function as a metabolic modulator by improving glucose tolerance and activating peroxisome proliferator-activated receptors (PPARs), which are crucial for managing obesity and type 2 diabetes [45]. In addition, phytol may demonstrate antiproliferative potential by inducing apoptosis and protective autophagy in various cancer cell lines [45]. Regarding β-sitosterol and stigmasterol, they are the primary structural phytosterols in plant cell membranes, known for their ability to lower total and LDL cholesterol by competing for intestinal micelle absorption and enhancing trans-intestinal cholesterol excretion [46,47]. Beyond their hypolipidemic property, these phytosterols have also been shown to exhibit anti-inflammatory, antioxidant, and antimicrobial activities [46,47].

### 3.6. Antiproliferative Activity of the 50% EtOH Extracts and Acidified 50% EtOH Extracts on Cancer Cells

Table 5 shows the IC_50_ values of the 50% EtOH extracts and acidified 50% EtOH extracts of roselle leaves against the growth of BxPC-3 pancreatic cancer cells and A549 lung cancer cells. Results showed that the IC_50_ values of HS-6 (289.2 μg/mL) and HS-6A (326.9 μg/mL) were significantly lower than their corresponding Taitung No. 3 counterparts HS-3 (323.2 μg/mL) and HS-3A (356.8 μg/mL), indicating that the extracts of Taitung No. 6 exhibited stronger antiproliferative effects on BxPC-3 cells than those of Taitung No. 3. All roselle leaf extracts showed weak antiproliferative effects on A549 cells, and their IC_50_ values on A549 cells (about 2085–2251 μg/mL) were about seven-fold of those values on BxPC-3 cells. A consistent trend was observed for the positive control cisplatin (a clinical anticancer drug), with the IC_50_ values of about 0.5 and 4.2 μg/mL on BxPC-3 and A549 cells, respectively, suggesting that A549 cells may possess higher intrinsic resistance to the anticancer agent in the experimental model used in this study.

**Figure 6:**
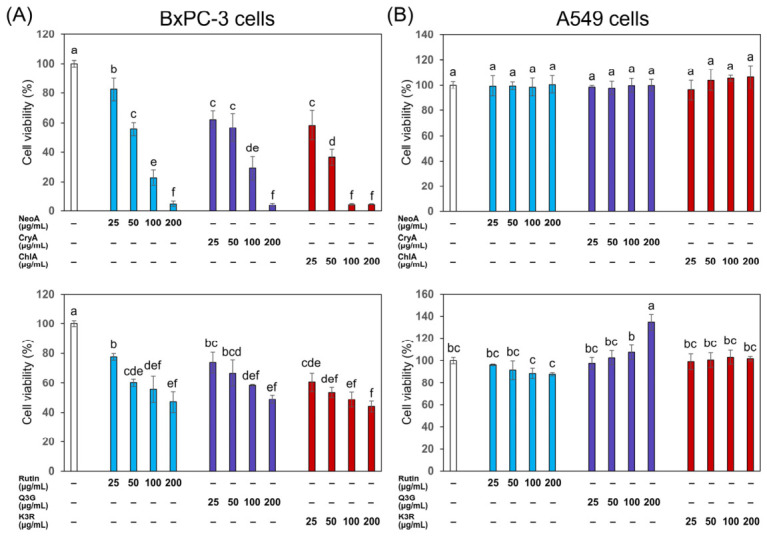
The inhibitory effects of neochlorogenic acid (NeoA), cryptochlorogenic acid (CryA), chlorogenic acid (ChlA), rutin, quercetin-3-*O*-glucoside (Q3G), and kaempferol-3-*O*-rutinoside (K3R) on the proliferation of (**A**) BxPC-3 pancreatic cancer cells and (**B**) A549 lung cancer cells at concentrations ranging from 25 to 200 μg/mL. Cell viability was normalized to the untreated control group (set as 100%). Data are presented as mean ± SD (*n* = 3). Different letters above the bars indicate significant differences between groups (*p* < 0.05) as determined by one-way ANOVA and then Tukey’s HSD test.

In order to identify the active compounds responsible for the observed antiproliferative activity in the samples, both top three abundant phenolic acids (including neochlorogenic acid, cryptochlorogenic acid, and chlorogenic acid) and flavonoids (including rutin, quercetin-3-*O*-glucoside, and kaempferol-3-*O*-rutinoside) in the extracts of roselle leaves were selected for antiproliferative assays (Figure 6). The results showed that all selected compounds exhibited dose-dependent inhibition on the growth of BxPC-3 cells (Figure 6A), and the chlorogenic acids possessed a stronger antiproliferative potency (with IC_50_ values lower than 100 μg/mL) than those of the flavonoid compounds (with IC_50_ values higher than 100 μg/mL). Therefore, these selected phenolic acids and flavonoids may serve as the primary active compounds contributing to the antiproliferative activity against BxPC-3 cells. This finding was consistent with the observed potency trend, where HS-6 and HS-3 (containing higher amounts of these active compounds) possessed significantly lower IC_50_ values than their acidified counterparts HS-6A and HS-3A. However, none of the six selected compounds exhibited a significant inhibitory effect on A549 cell proliferation.

Previous studies have revealed that chlorogenic acid exerts potent anticancer activity by arresting the cell cycle and promoting apoptosis, and it has been shown to suppress pancreatic carcinoma by inhibiting cellular bioenergetics (energy expenditure) through modulating the cellular-myelocytomatosis oncogene-transferrin receptor 1 (c-Myc-TFR1) axis [48,49]. Furthermore, quercetin exhibited significant anticancer potential through the inhibition of cancer cell proliferation and the induction of apoptosis and autophagy [50]. Kaempferol has also been shown to possess multiple anticancer effects, and it can inhibit pancreatic cancer cell growth and migration by targeting the phosphatidylinositol 3-kinase/protein kinase B (PI3K/AKT) signaling and epidermal growth factor receptor (EGFR)-related pathways [51]. In addition, studies have demonstrated that the combination of flavonoids such as quercetin and kaempferol exhibited stronger antiproliferative effects than single compounds in cancer cells [51,52]. These findings may explain the limited antiproliferative effects on A549 cells when the selected compounds were tested individually in this study. It is possible that synergistic interactions exist among the potential active compounds, or that other candidate compounds present in the roselle leaf extracts may contribute collectively to the inhibition of A549 cell growth. Anthocyanins may serve as potential contributors to the suppression of cancer cell growth because HS-6 and HS-6A (containing significantly higher amounts of anthocyanins) exhibited relatively stronger antiproliferative activity compared to HS-3 and HS-3A. Previous studies have reported that anthocyanins can inhibit cancer cell growth and induce apoptosis by downregulating the PI3K/AKT/mTOR, mitogen-activated protein kinase (MAPK), and Wnt/β-catenin signaling pathways [53,54].

### 3.7. Insulin Resistance-Ameliorating Activity of the 50% EtOH Extracts and Acidified 50% EtOH Extracts

**Figure 7:**
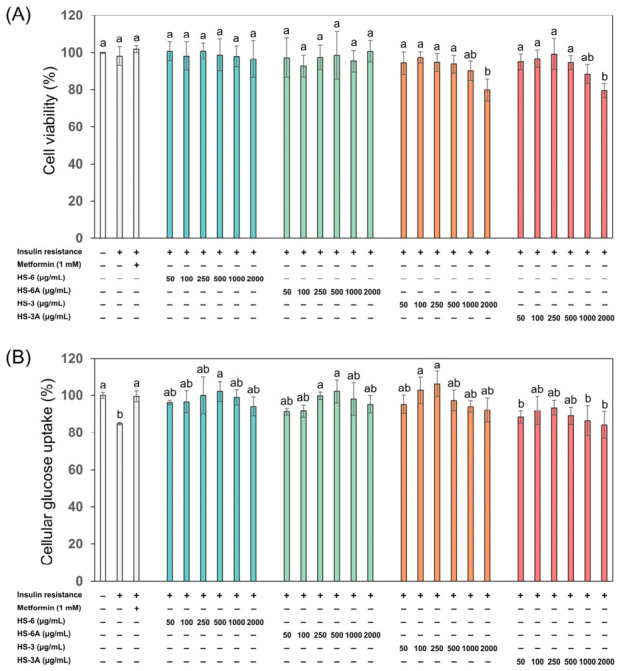
Assessment of glucose uptake in insulin-resistant (IR) HepG2 cells treated with the 50% EtOH extracts (HS-6 and HS-3) and acidified 50% EtOH extracts (HS-6A and HS-3A) of the leaves from Taitung No. 6 and Taitung No. 3 roselle at concentrations ranging from 50 to 2000 μg/mL. (**A**) Cell viability; (**B**) Cellular glucose uptake. Metformin was used as a positive control. Both cell viability and cellular glucose uptake were normalized to the untreated normal control group (without insulin resistance; set at 100%). Data are presented as mean ± SD (*n* = 3). Different letters above the bars indicate significant differences between groups (*p* < 0.05) as determined by one-way ANOVA and then Tukey’s HSD test.

The cell viability was first examined to ensure that the roselle leaf extracts were not toxic to the HepG2 cells used in the insulin-resistant (IR) model. Following preliminary concentration range-finding experiments, a wide concentration range of roselle extracts (50–2000 μg/mL) was applied to evaluate the potential toxicity on cells and the efficacy of improving cellular glucose uptake by alleviating insulin resistance. The results showed no significant change in the cell viability in the presence of roselle leaf extracts except at the highest concentration (2000 μg/mL) of HS-3 and HS-3A (Figure 7A), demonstrating that most of the samples did not affect the viability of HepG2 cells. In the IR cell model, the cellular glucose uptake decreased to about 85% compared to the untreated control group of normal cells (100%) (Figure 7B). However, treatments of HS-6 and HS-6A at concentrations of 50–500 μg/mL increased the glucose uptake in the IR cells, which was restored to approximately 100% at the concentrations of 250 and 500 μg/mL. The treatments of HS-3 exhibited a similar pattern in lower concentrations where the glucose uptake returned to about 100% and even higher at the concentrations of 100 and 250 μg/mL in the IR cells; nevertheless, HS-3A showed a relatively weak effect on improving glucose uptake, potentially due to the differences in its functional metabolite composition.

The results of decreased cellular glucose uptake indicated the induction of insulin resistance in the cells, whereas the increased glucose uptake in the presence of roselle leaf extracts indicated the amelioration of insulin resistance. Furthermore, it was observed that higher concentrations (such as 1000 and 2000 μg/mL) of roselle leaf extracts led to a relatively lower activity in the improvement of glucose uptake; this outcome may result from the biphasic dose–response relationship of phytochemicals, which exhibit beneficial effects at low doses and negative effects at higher doses due to over-stimulation or desensitization of specific biological pathways, leading to a diminished or even opposite response [55].

**Figure 8:**
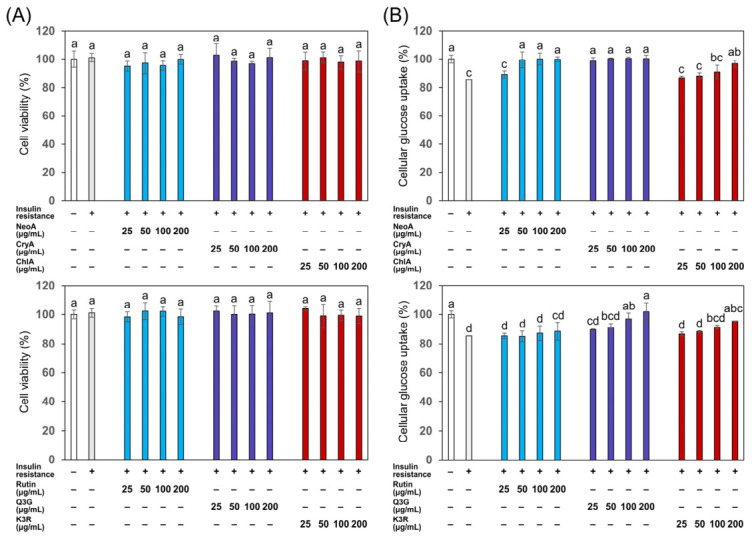
Assessment of glucose uptake in insulin-resistant (IR) HepG2 cells treated with neochlorogenic acid (NeoA), cryptochlorogenic acid (CryA), chlorogenic acid (ChlA), rutin, quercetin-3-*O*-glucoside (Q3G), and kaempferol-3-*O*-rutinoside (K3R) at concentrations ranging from 25 to 200 μg/mL. (**A**) Cell viability; (**B**) Cellular glucose uptake. Both cell viability and cellular glucose uptake were normalized to the untreated normal control group (without insulin resistance; set at 100%). Data are presented as mean ± SD (*n* = 3). Different letters above the bars indicate significant differences between groups (*p* < 0.05) as determined by one-way ANOVA and then Tukey’s HSD test.

The top three abundant phenolic acids and flavonoids in the roselle leaf extracts were further examined to identify the active compounds contributing to the improvement of insulin resistance. All six selected compounds showed no significant effect on the viability of cells at the concentrations of 25–200 μg/mL (Figure 8A). Neochlorogenic acid and cryptochlorogenic acid exhibited the most potent efficacy on ameliorating insulin resistance in the IR cells as demonstrated by the restoration of about 100% of the cellular glucose uptake at a relatively low concentration of 50 μg/mL (Figure 8B); the other four compounds (including chlorogenic acid, rutin, quercetin-3-*O*-glucoside, and kaempferol-3-*O*-rutinoside) showed dose-dependent improvement in the glucose uptake, with chlorogenic acid and quercetin-3-*O*-glucoside possessing a relatively stronger activity at the concentration of 200 μg/mL. These results demonstrated that the selected six compounds, especially neochlorogenic acid and cryptochlorogenic acid, may serve as the predominant active compounds for the insulin-sensitizing activity of the roselle leaf extracts.

Neochlorogenic acid has been reported to enhance insulin signaling by upregulating the phosphorylation of AKT and AMP-activated protein kinase (AMPK), thereby inducing the expression of insulin-responsive glucose transporter 4 (GLUT4) in insulin-resistant rats [56]. Studies have also revealed that chlorogenic acid may act as an insulin sensitizer by promoting glucose uptake through upregulating the expression of GLUT4 in diabetic rats and in patients with impaired glucose tolerance [57,58]. Flavonoids including rutin, quercetin, and kaempferol have been reported to possess insulin-sensitizing activity with similar mechanisms of action especially by activating the AKT or AMPK signaling pathways and enhancing the expression or membrane translocation of GLUT4 [59,60,61,62,63]. Moreover, synergistic attenuation of insulin resistance may occur among different phytochemicals such as chlorogenic acid and caffeic acid [64]. This suggests that there may be potential synergistic interactions contributing to the observed insulin resistance-ameliorating activity of roselle leaf extracts. However, the exact mechanisms of action remain to be elucidated in future studies.

## 4. Conclusions

This study established a metabolomic framework to profile the leaves of two Taiwanese roselle varieties, Taitung No. 6 and Taitung No. 3. Through integrated untargeted and targeted LC-MS/MS and GC-MS analyses, the distinct chemical features of the 50% EtOH extracts were characterized by high levels of neochlorogenic acid, cryptochlorogenic acid, rutin, and nicotiflorin, with the Taitung No. 6 variety exhibiting a specific enrichment of anthocyanins (D3S and C3S). Furthermore, the application of acidified extraction was shown to alter the metabolite profile by increasing the concentrations of flavonoid aglycones and polyamines. The *n*-hexane extracts of roselle leaves may serve as potential sources of lipophilic functional components, including vitamin E, α-linolenic acid, squalene, and phytol. Bioactivity evaluations suggested that the 50% EtOH leaf extracts of both varieties exhibited antiproliferative activity against BxPC-3 cells and were effective in ameliorating insulin resistance in HepG2 cells. Among the identified metabolites, neochlorogenic acid and cryptochlorogenic acid may serve as the major active compounds contributing to these bioactivities. Overall, these findings highlighted the potential of Taiwanese roselle leaves as a promising source of diverse bioactive compounds, and further animal studies and clinical trials are warranted to validate their efficacy and safety. Future research should also focus on product development and formulation stability to facilitate the industrial utilization and valorization of roselle leaves as a high-value agricultural by-product.

## Figures and Tables

**Table 1:** Extraction yields of different extracts of roselle leaves.

	Extraction Yield (%)		
Variety	50% EtOH Extract	Acidified 50% EtOH Extract	Hexane Extract
Taitung No. 6	29.67 ± 1.13 ^b,A^	33.39 ± 1.28 ^a,A^	5.20 ± 0.20 ^c,A^
Taitung No. 3	30.81 ± 1.14 ^a,A^	32.15 ± 0.86 ^a,A^	5.53 ± 0.08 ^b,A^

Values are mean ± SD (*n* = 3). The percentage of extraction yield is on a dry weight basis. Extraction yield (%) = [(weight of extract)/(weight of original dried leaf powder)] × 100%. Values in the same row with different lowercase letters are significantly different (*p* < 0.05) among the three extracts as determined by one-way ANOVA and then Tukey’s HSD test. Values in the same column with the same uppercase letter are not significantly different (*p* < 0.05) between the two roselle varieties as determined by Student’s *t*-test.

**Table 2:** Contents of total phenolics, flavonoids, and anthocyanins, and DPPH free radical scavenging activity of the 50% EtOH extracts (HS-6 and HS-3) and acidified 50% EtOH extracts (HS-6A and HS-3A) of the leaves from Taitung No. 6 and Taitung No. 3 roselle.

Extract	TPC (mg GAE/g)	TFC (mg RE/g)	TAC (mg D3SE/g)	DPPH EC_50_ (μg/mL)
HS-6	62.95 ± 0.80 ^a^	90.76 ± 3.61 ^b^	1.23 ± 0.08 ^a^	168.9 ± 8.2 ^b^
HS-6A	60.68 ± 0.99 ^b^	78.60 ± 3.33 ^c^	1.34 ± 0.06 ^a^	165.3 ± 13.4 ^b^
HS-3	61.50 ± 1.69 ^ab^	101.03 ± 4.37 ^a^	ND	171.9 ± 15.1 ^b^
HS-3A	60.23 ± 1.26 ^b^	88.01 ± 6.11 ^b^	ND	198.3 ± 7.8 ^a^

Values are mean ± SD (*n* = 3). ND: not detected. TPC: total phenolic content; TFC: total flavonoid content; TAC: total anthocyanin content. GAE: gallic acid equivalent; RE: rutin equivalent; D3SE: delphinidin-3-*O*-sambubioside equivalent. DPPH EC_50_: the effective concentration of the sample required to scavenge 50% of the DPPH free radical. TPC, TFC, and TAC values are expressed on a dry weight basis. For TPC, TFC, and DPPH EC_50_, values in the same column with different letters are significantly different (*p* < 0.05) as determined by two-way ANOVA and then Tukey’s HSD test. For TAC, values in the same column with the same letter are not significantly different (*p* < 0.05) as determined by Student’s *t*-test.

**Table 3:** Contents of the characteristic phenolic compounds in the 50% EtOH extracts (HS-6 and HS-3) and acidified 50% EtOH extracts (HS-6A and HS-3A) of the leaves from Taitung No. 6 and Taitung No. 3 roselle.

		Content (mg/g Extract)			
		Taitung No. 6		Taitung No. 3	
Compound	RT (min)	HS-6	HS-6A	HS-3	HS-3A
Compounds detected in positive ion mode					
*Anthocyanins*					
Delphinidin-3-*O*-sambubioside (D3S)	5.95	0.900 ± 0.113 ^a^	0.981 ± 0.223 ^a^	0.073 ± 0.007 ^b^	0.076 ± 0.004 ^b^
Delphinidin-3-*O*-glucoside (D3G)	5.96	0.057 ± 0.008 ^a^	0.063 ± 0.003 ^a^	Tr	0.005 ± 0.001 ^b^
Cyanidin-3-*O*-glucoside (C3G)	6.85	0.018 ± 0.002 ^a^	0.019 ± 0.001 ^a^	ND	ND
Cyanidin-3-*O*-sambubioside (C3S)	7.04	0.289 ± 0.002 ^a^	0.320 ± 0.095 ^a^	0.023 ± 0.002 ^b^	0.023 ± 0.001 ^b^
Compounds detected in negative ion mode					
*Phenolic acids*					
Shikimic acid	0.77	0.039 ± 0.012 ^a^	0.037 ± 0.007 ^a^	0.031 ± 0.003 ^a^	0.031 ± 0.003 ^a^
Gallic acid	2.40	0.005 ± 0.001 ^a^	0.007 ± 0.001 ^a^	Tr	Tr
Protocatechuic acid	3.85	0.016 ± 0.001 ^c^	0.017 ± 0.001 ^c^	0.031 ± 0.001 ^a^	0.027 ± 0.002 ^b^
Neochlorogenic acid	4.85	6.461 ± 0.374 ^a^	5.037 ± 0.493 ^bc^	5.637 ± 0.199 ^bc^	4.718 ± 0.511 ^c^
4-Hydroxybenzoic acid	5.73	Tr	Tr	0.006 ± 0.001 ^a^	0.007 ± 0.002 ^a^
Cryptochlorogenic acid	6.94	3.067 ± 0.118 ^b^	2.992 ± 0.068 ^b^	3.378 ± 0.089 ^a^	2.846 ± 0.181 ^b^
Syringic acid	7.35	0.047 ± 0.001 ^b^	0.063 ± 0.009 ^ab^	0.062 ± 0.004 ^ab^	0.069 ± 0.018 ^a^
Chlorogenic acid	7.37	0.242 ± 0.029 ^c^	0.220 ± 0.032 ^c^	0.345 ± 0.012 ^a^	0.299 ± 0.012 ^b^
Caffeic acid	7.47	Tr	0.014 ± 0.003 ^b^	0.079 ± 0.005 ^a^	0.083 ± 0.007 ^a^
*p*-Coumaric acid	9.85	Tr	Tr	0.009 ± 0.001 ^a^	0.009 ± 0.001 ^a^
Ferulic acid	10.18	0.007 ± 0.001 ^a^	0.008 ± 0.001 ^a^	0.007 ± 0.001 ^a^	0.006 ± 0.001 ^a^
Salicylic acid	10.93	0.018 ± 0.002 ^ab^	0.020 ± 0.003 ^a^	0.018 ± 0.001 ^ab^	0.016 ± 0.001 ^b^
*Flavonoids*					
Quercetin-3-*O*-rutinoside (rutin)	10.99	9.894 ± 0.667 ^bc^	8.899 ± 1.005 ^c^	12.819 ± 0.564 ^a^	10.893 ± 1.178 ^b^
Quercetin-3-*O*-glucoside (isoquercitrin)	11.36	2.879 ± 0.349 ^a^	2.211 ± 0.317 ^b^	0.877 ± 0.041 ^c^	0.661 ± 0.081 ^c^
Kaempferol-3-*O*-rutinoside (nicotiflorin)	11.73	2.873 ± 0.057 ^bc^	2.675 ± 0.425 ^c^	4.056 ± 0.133 ^a^	3.477 ± 0.382 ^b^
Kaempferol-3-*O*-glucoside (astragalin)	12.12	0.493 ± 0.018 ^a^	0.423 ± 0.092 ^a^	0.259 ± 0.013 ^b^	0.213 ± 0.023 ^b^
Myricetin	13.06	0.021 ± 0.004 ^a^	0.026 ± 0.005 ^a^	0.008 ± 0.001 ^b^	0.009 ± 0.001 ^b^
Quercetin	14.65	0.259 ± 0.029 ^b^	0.443 ± 0.069 ^a^	0.235 ± 0.035 ^b^	0.300 ± 0.081 ^b^
Kaempferol	16.13	0.062 ± 0.005 ^b^	0.109 ± 0.020 ^a^	0.066 ± 0.007 ^b^	0.085 ± 0.019 ^ab^

Values are mean ± SD (*n* = 3) on a dry weight basis. RT: retention time; ND: not detected; Tr: trace (<0.005 mg/g extract). Values in the same row with different letters are significantly different (*p* < 0.05) as determined by two-way ANOVA and then Tukey’s HSD test.

**Table 4:** Compounds in the hexane extracts of the leaves of Taitung No. 6 and Taitung No. 3 roselle.

		Relative Peak Area (%)	
Compound	RT (min)	Taitung No. 6	Taitung No. 3
*Fatty acids*			
Myristic acid (C14:0), TMS derivative	9.80	0.39 ± 0.01	0.50 ± 0.03
Palmitic acid (C16:0), TMS derivative	12.97	13.42 ± 1.01	15.92 ± 0.60
Heptadecanoic acid (C17:0), TMS derivative	15.30	0.28 ± 0.01	0.37 ± 0.01
Linoleic acid (C18:2), TMS derivative	17.14	3.44 ± 0.08	3.14 ± 0.13
α-Linolenic acid (C18:3), TMS derivative	17.31	12.59 ± 0.21	11.44 ± 0.58
Oleic acid (C18:1), TMS derivative	17.51	0.18 ± 0.01	0.20 ± 0.01
Stearic acid (C18:0), TMS derivative	18.03	2.51 ± 0.45	3.93 ± 0.04
Arachidic acid (C20:0), TMS derivative	21.44	0.25 ± 0.01	0.57 ± 0.06
Behenic acid (C22:0), TMS derivative	23.66	0.29 ± 0.01	0.52 ± 0.06
Tricosanoic acid (C23:0), TMS derivative	24.58	0.20 ± 0.01	0.32 ± 0.02
Lignoceric acid (C24:0), TMS derivative	25.42	0.45 ± 0.02	0.70 ± 0.03
Pentacosanoic acid (C25:0), TMS derivative	26.19	0.19 ± 0.02	0.25 ± 0.01
Hexacosanoic acid (C26:0), TMS derivative	27.02	0.19 ± 0.01	0.24 ± 0.01
*Terpenoids*			
Neophytadiene	9.73	0.77 ± 0.03	0.61 ± 0.03
Phytol, TMS derivative	16.08	8.23 ± 0.16	3.59 ± 0.15
Squalene	25.25	8.34 ± 0.29	4.90 ± 0.20
Lupeol, TMS derivative	30.65	0.66 ± 0.06	Tr
α-Amyrin, TMS derivative	30.81	1.61 ± 0.01	1.45 ± 0.04
24-Norursa-3,12-diene	31.62	0.57 ± 0.01	1.47 ± 0.14
*Steroids*			
Campesterol, TMS derivative	29.31	1.10 ± 0.01	1.09 ± 0.01
Stigmasterol, TMS derivative	29.61	2.75 ± 0.04	2.48 ± 0.09
β-Sitosterol, TMS derivative	30.42	3.83 ± 0.05	3.70 ± 0.01
*Alkanes*			
Tetracosane	24.27	0.19 ± 0.01	0.12 ± 0.02
Nonacosane	25.94	1.04 ± 0.03	0.67 ± 0.07
Dotriacontane	26.74	0.27 ± 0.01	0.25 ± 0.01
Hexatriacontane	27.64	4.20 ± 0.20	4.85 ± 0.15
Tetratetracontane	28.68	0.77 ± 0.01	0.99 ± 0.07
Tetrapentacontane	29.89	2.39 ± 0.08	3.65 ± 0.20
*Others*			
Lactic acid, 2TMS derivative	4.75	0.19 ± 0.01	0.24 ± 0.01
3-Hydroxy-2,3-dihydromaltol, 2TMS derivative	9.48	2.45 ± 0.02	1.51 ± 0.04
Tartronic acid, 3TMS derivative	10.76	0.56 ± 0.01	0.32 ± 0.03
7-Methoxy-3,7-dimethyloctan-1-ol, TMS derivative	12.76	6.66 ± 0.08	5.90 ± 0.35
1-Linolenoylglycerol, 2TMS derivative	24.76	0.21 ± 0.01	0.13 ± 0.01
*cis*-11-Eicosenamide	24.93	0.36 ± 0.01	0.28 ± 0.04
*n*-Heptadecan-1,2-diol, 2TMS derivative	26.48	0.21 ± 0.01	0.19 ± 0.01
γ-Tocopherol	27.20	0.13 ± 0.01	0.26 ± 0.02
α-Tocopherol	27.96	18.13 ± 0.94	23.22 ± 0.70

Values are mean ± SD (*n* = 3). RT: retention time; Tr: trace (<0.1%). Relative peak area (%) = [(chromatographic peak area of compound)/(the total area of all chromatographic peaks of the identified compounds)] × 100%.

**Table 5:** Inhibitory concentrations (IC_50_) of the 50% EtOH extracts (HS-6 and HS-3) and acidified 50% EtOH extracts (HS-6A and HS-3A) of the leaves from Taitung No. 6 and Taitung No. 3 roselle on BxPC-3 pancreatic cancer cell and A549 lung cancer cell growth.

	IC_50_ (μg/mL)	
Extract	BxPC-3 Cells	A549 Cells
HS-6	289.2 ± 6.8 ^c^	2084.7 ± 29.9 ^b^
HS-6A	326.9 ± 8.0 ^b^	2230.1 ± 98.1 ^a^
HS-3	323.2 ± 4.9 ^b^	2183.0 ± 33.1 ^ab^
HS-3A	356.8 ± 2.9 ^a^	2251.4 ± 24.7 ^a^
Cisplatin	0.5 ± 0.1 ^d^	4.2 ± 0.1 ^c^

Values are mean ± SD (*n* = 3). IC_50_: half-maximal inhibitory concentration (the concentration of samples that inhibit 50% of cell growth). Cisplatin was used as a positive control. Values in the same column with different letters are significantly different (*p* < 0.05) as determined by one-way ANOVA and then Tukey’s HSD test.

## Data Availability

The original contributions presented in the study are included in the article/Supplementary Materials. Further inquiries can be directed to the corresponding author.

## Supplementary Materials

The following supporting information can be downloaded at: https://www.mdpi.com/article/10.3390/foods15101696/s1, Table S1: The precursor ion, product ion, and collision energy conditions of anthocyanins in positive ion mode LC-triple quadrupole analysis, Table S2: The precursor ion, product ion, and collision energy conditions of phenolic acids and flavonoids in negative ion mode LC-triple quadrupole analysis, Table S3: Untargeted LC-MS/MS analysis results in positive ion mode, Table S4: The abbreviations for the original compound names used in the heatmap of positive ion mode data, Table S5: Untargeted LC-MS/MS analysis results in negative ion mode, Table S6: The abbreviations for the original compound names used in the heatmap of negative ion mode data.

## Abbreviations

The following abbreviations are used in this manuscript:

D3SE	Delphinidin-3-*O*-sambubioside equivalent
GAE	Gallic acid equivalent
GC-MS	Gas chromatography-mass spectrometry
HS-6	50% EtOH extract of Taitung No. 6 roselle leaves
HS-6A	Acidified 50% EtOH extract of Taitung No. 6 roselle leaves
HS-3	50% EtOH extract of Taitung No. 3 roselle leaves
HS-3A	Acidified 50% EtOH extract of Taitung No. 3 roselle leaves
IC_50_	Half-maximal inhibitory concentration
IR	Insulin resistant
LC-MS/MS	Liquid chromatography–tandem mass spectrometry
PCA	Principal component analysis
RE	Rutin equivalent
TAC	Total anthocyanin content
TFC	Total flavonoid content
TPC	Total phenolic content

## References

[1] Da-Costa-Rocha I., Bonnlaender B., Sievers H., Pischel I., Heinrich M. (2014). *Hibiscus sabdariffa* L.—A phytochemical and pharmacological review. Food Chem..

[2] Chew L.Y., Teng S.K., Neo Y.P., Sim Y.Y., Chew S.C. (2024). The potential of roselle (*Hibiscus sabdariffa*) plant in industrial applications: A promising source of functional compounds. J. Oleo Sci..

[3] Salem M.A., Zayed A., Beshay M.E., Abdel Mesih M.M., Ben Khayal R.F., George F.A., Ezzat S.M. (2022). *Hibiscus sabdariffa* L.: Phytoconstituents, nutritive, and pharmacological applications. Adv. Tradit. Med..

[4] Clímaco G.N., Vardanega R., Fasolin L.H. (2023). *Hibiscus sabdariffa* L. leaves as an alternative source of bioactive compounds obtained through high pressure technologies. J. Supercrit. Fluids.

[5] Zahari I., Ismail N., Johari M.S., Abdul Samad N. (2026). Optimisation of the extraction process and quality attributes of a roselle (*Hibiscus sabdariffa* L.) leaf tisane beverage. Processes.

[6] Kéllou K.K.B., Abdourazak M.A., Boukar M.C.M., Diagra M., Saley A.S., Yacoubou B. (2024). Roselle (*Hibiscus sabdariffa* L.): Overview of its biology, ecology, socio-economic importance and cultivation constraints in West Africa. World J. Pharm. Life Sci..

[7] Edo G.I., Samuel P.O., Jikah A.N., Oloni G.O., Ifejika M.N., Oghenegueke O., Ossai S., Ajokpaoghene M.O., Asaah E.U., Uloho P.O. (2023). Proximate composition and health benefit of Roselle leaf (*Hibiscus sabdariffa*). Insight on food and health benefits. Food Chem. Adv..

[8] Islam M. (2019). Food and medicinal values of roselle (*Hibiscus sabdariffa* L. Linne Malvaceae) plant parts: A review. Open J. Nutr. Food Sci..

[9] Lyu J.I., Kim J.M., Kim D.-G., Kim J.-B., Kim S.H., Ahn J.-W., Kang S.-Y., Ryu J., Kwon S.-J. (2020). Phenolic compound content of leaf extracts from different roselle (*Hibiscus sabdariffa*) accessions. Plant Breed. Biotech..

[10] Wang J., Cao X., Jiang H., Qi Y., Chin K.L., Yue Y. (2014). Antioxidant activity of leaf extracts from different *Hibiscus sabdariffa* accessions and simultaneous determination five major antioxidant compounds by LC-Q-TOF-MS. Molecules.

[11] Zhen J., Villani T.S., Guo Y., Qi Y., Chin K., Pan M.-H., Ho C.-T., Simon J.E., Wu Q. (2016). Phytochemistry, antioxidant capacity, total phenolic content and anti-inflammatory activity of *Hibiscus sabdariffa* leaves. Food Chem..

[12] Luthria D.L., Tareq F.S., Kotha R.R., Marupaka R., Harnly J.M., Arlotta C.G., Richardson M.L. (2021). Variation of phytochemicals in leaves of seven accessions of *Hibiscus sabdariffa* grown under field, green roof, and high tunnel conditions. ACS Food Sci. Technol..

[13] Rodríguez-Medina I.C., Beltrán-Debón R., Molina V.M., Alonso-Villaverde C., Joven J., Menéndez J.A., Segura-Carretero A., Fernández-Gutiérrez A. (2009). Direct characterization of aqueous extract of *Hibiscus sabdariffa* using HPLC with diode array detection coupled to ESI and ion trap MS. J. Sep. Sci..

[14] Delfin M.M., Marutani M. (2025). Studies on phytochemistry and antioxidant capacity of nine *Hibiscus sabdariffa* accessions. HortScience.

[15] Al-Wandawi H. (2015). Organic acids composition of different parts of the medicinal plant–roselle (*Hibiscus sabdariffa*). Int. J. Biol. Pharm. Res..

[16] Bhandare S.D., Malode S.S. (2024). Examination of *Hibiscus sabdariffa*’s bioactive arsenal: Pharmacological prospects and comprehensive insights into catalysing health and wellness through its therapeutic attributes—An exposition. J. Pharm. Pharmacol..

[17] Muhamad Rosli S.H., Lim X.Y., Krishnan P., Ahmad I.F., Voon Y.L., Siau T.C., Chan J.S.W., Omar M.H., Tan T.Y.C. (2026). Unveiling the safety, tolerability, and herb-drug interaction concerns of *Hibiscus sabdariffa* L. (Roselle): A systematic scoping review of current evidence. Heliyon.

[18] Ochani P.C., D’Mello P. (2009). Antioxidant and antihyperlipidemic activity of *Hibiscus sabdariffa* Linn. leaves and calyces extracts in rats. Indian J. Exp. Biol..

[19] Ajiboye B.O., Famusiwa C.D., Oyedare D.I., Paul B., Julius Z.O.A., Ojo O.A., Akindele A.F.I., Hosseinzadeh H., Brai B.I., Oyinloye B.E. (2024). *Hibiscus sabdariffa* leaf extract enhances molecular gene expression of insulin and GLP-1 receptors in streptozotocin-induced rats. Spec. J. Pharmacogn. Phytochem. Biotechnol..

[20] Ndarubu T.A., Chiamaka O.S., Alfa S., Aishatu M., Chinedu O., Wenawo D., Eustace B. (2019). Phytochemicals, hypoglycemic and hypolipidemic effects of methanol leaf extract of *Hibiscus sabdariffa* in alloxan induced diabetic rats. GSC Biol. Pharm. Sci..

[21] Lin H.-H., Chan K.-C., Sheu J.-Y., Hsuan S.-W., Wang C.-J., Chen J.-H. (2012). *Hibiscus sabdariffa* leaf induces apoptosis of human prostate cancer cells in vitro and in vivo. Food Chem..

[22] Chiu C.-T., Hsuan S.-W., Lin H.-H., Hsu C.-C., Chou F.-P., Chen J.-H. (2015). *Hibiscus sabdariffa* leaf polyphenolic extract induces human melanoma cell death, apoptosis, and autophagy. J. Food Sci..

[23] Chen J.-W., Chen Y.-F. (2022). A new high-anthocyanin roselle variety “Taitung No. 6-Black Crystal”. Agric. Policy Rev..

[24] Chen J.-W. (2022). Roselle new variety Taitung No. 6–Black Crystal. Taitung Dist. Agric. Tech. Bull..

[25] Characteristic Diagram of Taitung No. 3 Variety. https://kmweb.moa.gov.tw/subject/subject.php?id=26453.

[26] Taiwan Good Agriculture Practice (TGAP) for Roselle. https://drive.google.com/uc?export=download&id=1tbyoVTz3zSIBrW5q1MZ7pcwS0SkMMpDX.

[27] Yang D., Li M.-M., Wang W.-J., Zheng G.-D., Yin Z.-P., Chen J.-G., Zhang Q.-F. (2022). Separation and purification of anthocyanins from Roselle by macroporous resins. LWT-Food Sci. Technol..

[28] Singleton V.L., Rossi J.A. (1965). Colorimetry of total phenolics with phosphomolybdic-phosphotungstic acid reagents. Am. J. Enol. Vitic..

[29] Wu S.-J., Ng L.-T. (2008). Antioxidant and free radical scavenging activities of wild bitter melon (*Momordica charantia* Linn. var. *abbreviata* Ser.) in Taiwan. LWT-Food Sci. Technol..

[30] Pang Z., Lu Y., Zhou G., Hui F., Xu L., Viau C., Spigelman A.F., MacDonald P.E., Wishart D.S., Li S. (2024). MetaboAnalyst 6.0: Towards a unified platform for metabolomics data processing, analysis and interpretation. Nucleic Acids Res..

[31] Piovesana A., Rodrigues E., Noreña C.P.Z. (2019). Composition analysis of carotenoids and phenolic compounds and antioxidant activity from hibiscus calyces (*Hibiscus sabdariffa* L.) by HPLC-DAD-MS/MS. Phytochem. Anal..

[32] Brand-Williams W., Cuvelier M.E., Berset C. (1995). Use of a free radical method to evaluate antioxidant activity. LWT-Food Sci. Technol..

[33] Pavasutti V., Sinthuvanich C., Tayana N., Kongkiatpaiboon S., Sae-tan S. (2023). Mung bean seed coat water extract restores insulin sensitivity via upregulation of antioxidant defense system and downregulation of inflammation in insulin-resistant HepG2 cells. NFS J..

[34] Wang M., Mao H., Chen J., Li Q., Ma W., Zhu N., Qi L., Wang J. (2022). Chinese bayberry (*Myrica rubra* Sieb. et Zucc.) leaves proanthocyanidins alleviate insulin-resistance via activating PI3K/AKT pathway in HepG2 cells. J. Funct. Foods.

[35] Hapsari B.W., Manikharda, Setyaningsih W. (2021). Methodologies in the analysis of phenolic compounds in roselle (*Hibiscus sabdariffa* L.): Composition, biological activity, and beneficial effects on human health. Horticulturae.

[36] Pękal A., Pyrzynska K. (2014). Evaluation of aluminium complexation reaction for flavonoid content assay. Food Anal. Methods.

[37] Singh S., Swain S., Singh D.R., Salim K.M., Nayak D., Roy S.D. (2015). Changes in phytochemicals, anti-nutrients and antioxidant activity in leafy vegetables by microwave boiling with normal and 5% NaCl solution. Food Chem..

[38] Ali H.M., Almagribi W., Al-Rashidi M.N. (2016). Antiradical and reductant activities of anthocyanidins and anthocyanins, structure–activity relationship and synthesis. Food Chem..

[39] Nuutila A.M., Kammiovirta K., Oksman-Caldentey K.M. (2002). Comparison of methods for the hydrolysis of flavonoids and phenolic acids from onion and spinach for HPLC analysis. Food Chem..

[40] Dawidowicz A.L., Typek R. (2011). The influence of pH on the thermal stability of 5-*O*-caffeoylquinic acids in aqueous solutions. Eur. Food Res. Technol..

[41] Mohamed R., Fernández J., Pineda M., Aguilar M. (2007). Roselle (*Hibiscus sabdariffa*) seed oil is a rich source of γ-tocopherol. J. Food Sci..

[42] Patted P.G., Masareddy R.S., Patil A.S., Kanabargi R.R., Bhat C.T. (2024). Omega-3 fatty acids: A comprehensive scientific review of their sources, functions and health benefits. Futur. J. Pharm. Sci..

[43] Fatima G., Dzupina A., Mahdi A.A., Fedacko J., Magomedova A., Yousif N.G. (2025). Role of vitamin-E as a vital nutrient in health and diseases. Indian J. Clin. Biochem..

[44] Lou-Bonafonte J.M., Martínez-Beamonte R., Sanclemente T., Surra J.C., Herrera-Marcos L.V., Sanchez-Marco J., Arnal C., Osada J. (2018). Current insights into the biological action of squalene. Mol. Nutr. Food Res..

[45] Islam M.T., Ali E.S., Uddin S.J., Shaw S., Islam M.A., Ahmed M.I., Chandra Shill M., Karmakar U.K., Yarla N.S., Khan I.N. (2018). Phytol: A review of biomedical activities. Food Chem. Toxicol..

[46] Li X., Xin Y., Mo Y., Marozik P., He T., Guo H. (2022). The bioavailability and biological activities of phytosterols as modulators of cholesterol metabolism. Molecules.

[47] Nattagh-Eshtivani E., Barghchi H., Pahlavani N., Barati M., Amiri Y., Fadel A., Khosravi M., Talebi S., Arzhang P., Ziaei R. (2022). Biological and pharmacological effects and nutritional impact of phytosterols: A comprehensive review. Phytother. Res..

[48] Nguyen V., Taine E.G., Meng D., Cui T., Tan W. (2024). Chlorogenic acid: A systematic review on the biological functions, mechanistic actions, and therapeutic potentials. Nutrients.

[49] Yang H., Said A.M., Huang H., Papa A.P.D., Jin G., Wu S., Ma N., Lan L., Shangguan F., Zhang Q. (2021). Chlorogenic acid depresses cellular bioenergetics to suppress pancreatic carcinoma through modulating c-Myc-TFR1 axis. Phytother. Res..

[50] Shahbaz M., Naeem H., Momal U., Imran M., Alsagaby S.A., Al Abdulmonem W., Waqar A.B., El-Ghorab A.H., Ghoneim M.M., Abdelgawad M.A. (2023). Anticancer and apoptosis inducing potential of quercetin against a wide range of human malignancies. Int. J. Food Prop..

[51] Amjad E., Sokouti B., Asnaashari S. (2022). A systematic review of anti-cancer roles and mechanisms of kaempferol as a natural compound. Cancer Cell Int..

[52] Ackland M.L., van de Waarsenburg S., Jones R. (2005). Synergistic antiproliferative action of the flavonols quercetin and kaempferol in cultured human cancer cell lines. In Vivo.

[53] Awad M.G., Hanafy N.A.N., Ali R.A., El-Monem D.D.A., El-Shafiey S.H., El-Magd M.A. (2026). Advances in anthocyanin research: From extraction to functional applications in health. Health Nanotechnol..

[54] Sharma A., Choi H.-K., Kim Y.-K., Lee H.-J. (2021). Delphinidin and its glycosides’ war on cancer: Preclinical perspectives. Int. J. Mol. Sci..

[55] Jodynis-Liebert J., Kujawska M. (2020). Biphasic dose-response induced by phytochemicals: Experimental evidence. J. Clin. Med..

[56] Singdam P., Naowaboot J., Senggunprai L., Boonloh K., Hipkaeo W., Pannangpetch P. (2023). The mechanisms of neochlorogenic acid (3-caffeoylquinic acid) in improving glucose and lipid metabolism in rats with insulin resistance induced by a high fat-high fructose diet. Trends Sci..

[57] Meng S., Cao J., Feng Q., Peng J., Hu Y. (2013). Roles of chlorogenic acid on regulating glucose and lipids metabolism: A review. Evid. Based Complement. Altern. Med..

[58] Zuñiga L.Y., Aceves-de la Mora M.C.A., González-Ortiz M., Ramos-Núñez J.L., Martínez-Abundis E. (2017). Effect of chlorogenic acid administration on glycemic control, insulin secretion, and insulin sensitivity in patients with impaired glucose tolerance. J. Med. Food.

[59] Arias N., Macarulla M.T., Aguirre L., Martínez-Castaño M.G., Portillo M.P. (2013). Quercetin can reduce insulin resistance without decreasing adipose tissue and skeletal muscle fat accumulation. Genes Nutr..

[60] Hsu C.-Y., Shih H.-Y., Chia Y.-C., Lee C.-H., Ashida H., Lai Y.-K., Weng C.-F. (2014). Rutin potentiates insulin receptor kinase to enhance insulin-dependent glucose transporter 4 translocation. Mol. Nutr. Food Res..

[61] Jiang Y., Wang L., Fan F., Fang Q., Li H., Wang M., Zhao Y. (2024). Rutin alleviates advanced glycosylation end products-induced insulin resistance by inhibiting SOCS3/IRS1 and activating PI3K/AKT signaling pathways in HepG2 cells. J. Funct. Foods.

[62] Russo B., Picconi F., Malandrucco I., Frontoni S. (2019). Flavonoids and insulin-resistance: From molecular evidences to clinical trials. Int. J. Mol. Sci..

[63] Santana-Lima B., Belaunde L.H.Z., de Souza K.D., Rosa M.E., de Carvalho J.E., Machado J., Alonso-Vale M.I.C., Caseli L., Rando D.G.G., Caperuto L.C. (2024). Acute kaempferol stimulation induces AKT phosphorylation in HepG2 cells. Life.

[64] Chen L., Teng H., Cao H. (2019). Chlorogenic acid and caffeic acid from *Sonchus oleraceus* Linn synergistically attenuate insulin resistance and modulate glucose uptake in HepG2 cells. Food Chem. Toxicol..

