# Supplementary material for: Metabolomic Profiling of Taiwanese Roselle (Hibiscus sabdariffa) Leaves and Their Antiproliferative and Insulin Resistance-Ameliorating Potential
Source: Foods. 2026 May 12;15(10):1696. doi: 10.3390/foods15101696 (PMC13206271; doi:10.3390/foods15101696)
Supplement: Supplementary file 1 [file foods-15-01696-s001.zip › foods-4274888-supplementary.pdf]

foods

MDPI

Metabolomic Profiling of Taiwanese Roselle (*Hibiscus sabdariffa*) Leaves and Their Antiproliferative and Insulin Resistance-Ameliorating Potential

Supplementary Materials

Table S1. The precursor ion, product ion, and collision energy conditions of anthocyanins in positive ion mode LC-triple quadrupole analysis.

Compound	RT (min)	*m/z* value		Collision energy (V)
		Precursor ion	Product ion	
Delphinidin-3-*O*-sambubioside	5.95	597.1	303.0 [a]<br>257.0	30<br>50
Delphinidin-3-*O*-glucoside	5.96	465.1	303.0 [a]<br>257.0	30<br>50
Cyanidin-3-*O*-glucoside	6.85	449.1	287.0 [a]<br>213.0	30<br>55
Cyanidin-3-*O*-sambubioside	7.04	581.2	287.0 [a]<br>213.0	30<br>60

[a] Product ion with higher signal intensity used for quantification.

Table S2. The precursor ion, product ion, and collision energy conditions of phenolic acids and flavonoids in negative ion mode LC-triple quadrupole analysis.

Compound	RT (min)	*m/z* value		Collision energy (V)
		Precursor ion	Product ion	
Shikimic acid	0.77	173.0	93.0 [a]	15
			137.0	15
Gallic acid	2.40	169.0	125.0 [a]	15
			107.0	15
Protocatechuic acid	3.85	153.0	109.0 [a]	15
			81.0	20
Neochlorogenic acid	4.85	353.1	191.0 [a]	25
			179.0	25
4-Hydroxybenzoic acid	5.73	137.0	93.0 [a]	20
			65.0	20
Cryptochlorogenic acid	6.94	353.1	173.0 [a]	20
			191.0	15
Syringic acid	7.35	197.0	123.0 [a]	20
			138.0	20
Chlorogenic acid	7.37	353.1	191.0 [a]	20
			135.0	45
Caffeic acid	7.47	179.0	135.1 [a]	20
			107.0	30
p-Coumaric acid	9.85	163.0	119.0 [a]	20
			93.0	30
Ferulic acid	10.18	193.1	134.0 [a]	20
			178.0	15
Salicylic acid	10.93	137.0	93.1 [a]	20
			65.2	30
Quercetin-3-*O*-rutinoside (rutin)	10.99	609.1	300.0 [a]	35
			271.0	60
Quercetin-3-*O*-glucoside (isoquercitrin)	11.36	463.1	300.0 [a]	30
			271.0	55
Kaempferol-3-*O*-rutinoside (nicotiflorin)	11.73	593.2	285.0 [a]	35
			255.0	60
Kaempferol-3-*O*-glucoside (astragalin)	12.12	447.1	284.0 [a]	30
			255.0	45
Myricetin	13.06	317.0	151.0 [a]	25
			137.0	25
Quercetin	14.65	301.0	151.0 [a]	20
			179.0	20
Kaempferol	16.13	285.0	117.0 [a]	45
			93.0	45

[a] Product ion with higher signal intensity used for quantification.

Table S3. Untargeted LC-MS/MS analysis results in positive ion mode.

Compound name	RT (min)	Ion adduct	Molecular formula	Observed *m/z*	Theoretical *m/z*	*m/z* diff (ppm)	Similarity score	Relative peak area (%)			
								HS-6	HS-6A	HS-3	HS-3A
Spermidine	0.5750	$[M+H]^+$	$C_7H_{19}N_3$	146.1656	146.1652	2.48	0.979	0.761 ± 0.008	1.003 ± 0.016	0.399 ± 0.007	0.648 ± 0.002
Agmatine	0.5826	$[M+H]^+$	$C_5H_{14}N_4$	131.1296	131.1291	3.81	0.850	0.946 ± 0.041	0.957 ± 0.024	1.010 ± 0.006	1.051 ± 0.003
Choline	0.6353	$[M]^+$	$[C_5H_{14}NO]^+$	104.1074	104.1075	-1.78	0.920	5.831 ± 0.052	6.198 ± 0.109	7.945 ± 0.040	7.839 ± 0.057
γ-Aminobutyric acid	0.6507	$[M+H]^+$	$C_4H_9NO_2$	104.0704	104.0706	-1.70	0.823	0.833 ± 0.021	0.876 ± 0.020	0.903 ± 0.005	0.918 ± 0.011
Asparagine	0.6779	$[M+H]^+$	$C_4H_8N_2O_3$	133.0608	133.0608	0.06	0.964	0.105 ± 0.003	0.106 ± 0.004	Tr	Tr
Phosphorylcholine	0.7021	$[M+H]^+$	$C_5H_{14}NO_4P$	184.0733	184.0733	-0.11	0.978	0.188 ± 0.003	0.214 ± 0.016	0.293 ± 0.004	0.305 ± 0.001
Betaine	0.7028	$[M+H]^+$	$C_5H_{11}NO_2$	118.0864	118.0863	1.09	0.993	20.987 ± 0.173	22.967 ± 0.505	15.239 ± 0.367	15.405 ± 0.427
Trigonelline	0.7213	$[M+H]^+$	$C_7H_7NO_2$	138.0549	138.0550	-0.41	0.969	Tr	0.100 ± 0.004	0.117 ± 0.005	0.122 ± 0.004
Proline	0.7247	$[M+H]^+$	$C_5H_9NO_2$	116.0703	116.0706	-2.23	0.985	1.171 ± 0.015	1.270 ± 0.003	1.261 ± 0.004	1.284 ± 0.004
sn-Glycero-3-phosphocholine	0.7263	$[M+H]^+$	$C_8H_{20}NO_6P$	258.1103	258.1101	0.66	0.929	0.236 ± 0.012	0.250 ± 0.008	1.020 ± 0.008	0.968 ± 0.005
Adenine	0.7500	$[M+H]^+$	$C_5H_5N_5$	136.0614	136.0618	-2.66	0.999	0.308 ± 0.012	0.396 ± 0.011	0.560 ± 0.009	0.634 ± 0.007
3-Hydroxy-2-methyl-4-pyrone or isomer_1	0.7544	$[M+H]^+$	$C_6H_6O_3$	127.0382	127.0390	-6.03	0.987	0.703 ± 0.007	0.262 ± 0.001	0.891 ± 0.001	0.709 ± 0.017
Guanine	0.7778	$[M+H]^+$	$C_5H_5N_5O$	152.0563	152.0570	-4.48	0.904	Tr	Tr	0.192 ± 0.004	0.192 ± 0.003
Pyridoxine	0.7939	$[M+H]^+$	$C_8H_{11}NO_3$	170.0813	170.0813	0.08	0.982	Tr	0.105 ± 0.002	0.142 ± 0.002	0.141 ± 0.001
Isoleucine	0.8024	$[M+H]^+$	$C_6H_{13}NO_2$	132.1022	132.1026	-3.27	0.990	5.429 ± 0.008	5.125 ± 0.030	4.350 ± 0.047	4.327 ± 0.020
Cuminyl alcohol	0.8036	$[M+H-H_2O]^+$	$C_{10}H_{14}O$	133.1025	133.1010	11.61	0.862	0.309 ± 0.003	0.308 ± 0.006	0.232 ± 0.003	0.232 ± 0.015
Pyroglutamic acid	1.1485	$[M+H]^+$	$C_5H_7NO_3$	130.0492	130.0499	-4.92	0.959	Tr	Tr	0.144 ± 0.004	0.140 ± 0.003
Tyrosine	1.1743	$[M+H]^+$	$C_9H_{11}NO_3$	182.0813	182.0812	0.48	0.992	0.301 ± 0.008	0.300 ± 0.004	0.269 ± 0.007	0.270 ± 0.005
Adenosine	1.1856	$[M+H]^+$	$C_{10}H_{13}N_5O_4$	268.1043	268.1040	1.00	0.995	0.783 ± 0.021	0.413 ± 0.015	0.610 ± 0.005	0.404 ± 0.003
Phenylalanine	1.7495	$[M+H]^+$	$C_9H_{11}NO_2$	166.0869	166.0863	4.04	0.991	16.873 ± 0.528	16.959 ± 0.615	12.938 ± 0.073	13.071 ± 0.098
3-Hydroxy-2-methyl-4-pyrone or isomer_2	2.0113	$[M+H]^+$	$C_6H_6O_3$	127.0383	127.0390	-5.17	0.989	Tr	0.159 ± 0.004	0.103 ± 0.006	0.223 ± 0.002
Indoleacrylic acid	2.7156	$[M+H]^+$	$C_{11}H_9NO_2$	188.0704	188.0706	-1.25	0.829	1.643 ± 0.050	0.887 ± 0.020	0.142 ± 0.001	0.145 ± 0.003

Table S3 (continued). Untargeted LC-MS/MS analysis results in positive ion mode.

Compound name	RT (min)	Ion adduct	Molecular formula	Observed *m/z*	Theoretical *m/z*	*m/z* diff (ppm)	Similarity score	Relative peak area (%)			
								HS-6	HS-6A	HS-3	HS-3A
Tryptophan	2.7169	$[M+H]^+$	$C_{11}H_{12}N_2O_2$	205.0976	205.0972	1.99	0.972	2.916 ± 0.122	1.632 ± 0.093	0.270 ± 0.022	0.269 ± 0.016
5'-*S*-Methylthioadenosine	2.8022	$[M+H]^+$	$C_{11}H_{15}N_5O_3S$	298.0964	298.0968	-1.50	0.993	0.644 ± 0.004	0.356 ± 0.010	Tr	Tr
N^6-Succinyladenosine	2.9014	$[M+H]^+$	$C_{14}H_{17}N_5O_8$	384.1149	384.1150	-0.25	0.874	0.120 ± 0.006	Tr	0.182 ± 0.003	Tr
Rosmarinic acid	4.0774	$[M+H-C_9H_{10}O_5]^+$	$C_{18}H_{16}O_8$	163.0390	163.0397	-4.31	0.897	4.941 ± 0.105	4.862 ± 0.037	5.340 ± 0.020	5.416 ± 0.068
Chlorogenic acid	4.0782	$[M+H]^+$	$C_{16}H_{18}O_9$	355.1031	355.1031	0.27	0.984	2.975 ± 0.037	2.979 ± 0.071	3.205 ± 0.025	3.250 ± 0.008
Caffeic acid	4.0793	$[M+H]^+$	$C_9H_8O_4$	181.0492	181.0501	-4.80	0.951	0.166 ± 0.007	0.176 ± 0.009	0.220 ± 0.002	0.231 ± 0.004
Tetrahydroharman-3-carboxylic acid	4.3954	$[M+H]^+$	$C_{13}H_{14}N_2O_2$	231.1127	231.1128	-0.48	0.944	0.282 ± 0.011	0.329 ± 0.022	0.785 ± 0.003	0.807 ± 0.005
(*E*)-3-[4-[(2*S*,3*R*,4*S*,5*S*,6*R*)-3,4,5-trihydroxy-6-(hydroxymethyl)oxan-2-yl]oxyphenyl]prop-2-enoic acid (*p*-coumaric acid-4-O-glucoside)	4.7402	$[M+NH_4]^+$	$C_{15}H_{18}O_8$	344.1343	344.1340	0.86	0.891	0.531 ± 0.012	0.697 ± 0.019	0.978 ± 0.017	1.091 ± 0.021
2-(3,4-dihydroxyphenyl)-5,7-dihydroxy-3-[(2*S*,3*R*,4*S*,5*S*,6*R*)-3,4,5-trihydroxy-6-[[(2*S*,3*R*,4*S*,5*R*)-3,4,5-trihydroxyoxan-2-yl]oxymethyl]oxan-2-yl]oxychromen-4-one (quercetin-3-*O*-vicianoside) or isomer_1	4.9298	$[M+H]^+$	$C_{26}H_{28}O_{16}$	597.1439	597.1450	-1.93	0.871	0.114 ± 0.013	0.209 ± 0.013	Tr	Tr
Coumarin	5.4161	$[M+H]^+$	$C_9H_6O_2$	147.0440	147.0441	-0.64	0.914	0.184 ± 0.003	0.212 ± 0.016	0.234 ± 0.005	0.253 ± 0.000
5,7-Dihydroxy-2-(4-hydroxyphenyl)-3-[(2*S*,3*R*,4*S*,5*S*,6*R*)-3,4,5-trihydroxy-6-[[(2*S*,3*R*,4*S*,5*S*)-3,4,5-trihydroxyoxan-2-yl]oxymethyl]oxan-2-yl]oxychromen-4-one (kaempferol-3-*O*-vicianoside) or isomer_1	6.0579	$[M+H]^+$	$C_{26}H_{28}O_{15}$	581.1492	581.1501	-1.57	0.922	0.329 ± 0.005	0.317 ± 0.012	Tr	Tr
Roseoside	7.9870	$[M+H]^+$	$C_{19}H_{30}O_8$	387.2005	387.2030	-6.35	0.846	Tr	Tr	0.350 ± 0.008	0.338 ± 0.002
Loliolide	8.2488	$[M+H]^+$	$C_{11}H_{16}O_3$	197.1178	197.1170	4.05	0.799	1.717 ± 0.024	1.721 ± 0.032	2.040 ± 0.033	2.046 ± 0.020

Table S3 (continued). Untargeted LC-MS/MS analysis results in positive ion mode.

Compound name	RT (min)	Ion adduct	Molecular formula	Observed *m/z*	Theoretical *m/z*	*m/z* diff (ppm)	Similarity score	Relative peak area (%)			
								HS-6	HS-6A	HS-3	HS-3A
3-[6-[[(2*R*,3*R*,4*R*,5*S*,6*S*)-3,5-dihydroxy-6-methyl-4-[(2*S*,3*R*,4*R*,5*R*,6*S*)-3,4,5-trihydroxy-6-methyloxan-2-yl]oxyoxan-2-yl]oxymethyl]-3,4,5-trihydroxyoxan-2-yl]oxy-2-(3,4-dihydroxyphenyl)-5,7-dihydroxychromen-4-one (quercetin-3-*O*-glucopyranosyl-dirhamnopyranoside) or isomer_1	9.3171	$[M+H]^+$	$C_{33}H_{40}O_{20}$	757.2187	757.2186	0.07	0.974	0.101 ± 0.003	Tr	0.120 ± 0.000	0.121 ± 0.000
2-(3,4-dihydroxyphenyl)-5,7-dihydroxy-3-[(2*S*,3*R*,4*S*,5*S*,6*R*)-3,4,5-trihydroxy-6-[[(2*S*,3*R*,4*S*,5*R*)-3,4,5-trihydroxyoxan-2-yl]oxymethyl]oxan-2-yl]oxychromen-4-one (quercetin-3-*O*-vicianoside) or isomer_2	9.7036	$[M+H]^+$	$C_{26}H_{28}O_{16}$	597.1444	597.1450	-1.01	0.913	0.344 ± 0.015	0.134 ± 0.021	Tr	Tr
3-[(2*S*,3*R*,4*S*,5*R*,6*R*)-6-[[(2*R*,3*R*,4*R*,5*S*,6*S*)-3,5-dihydroxy-6-methyl-4-[(2*S*,3*R*,4*R*,5*R*,6*S*)-3,4,5-trihydroxy-6-methyloxan-2-yl]oxyoxan-2-yl]oxymethyl]-3,4,5-trihydroxyoxan-2-yl]oxy-5,7-dihydroxy-2-(4-hydroxyphenyl)chromen-4-one (kaempferol-3-*O*-glucopyranosyl-dirhamnopyranoside) or isomer_1	9.8247	$[M+H]^+$	$C_{33}H_{40}O_{19}$	741.2239	741.2237	0.29	0.978	0.113 ± 0.004	0.105 ± 0.001	0.114 ± 0.000	0.115 ± 0.001
Quercetin or isomer_1	10.0165	$[M+H]^+$	$C_{15}H_{10}O_7$	303.0508	303.0499	2.83	0.970	0.419 ± 0.006	0.424 ± 0.010	1.153 ± 0.054	1.253 ± 0.003
Rutin	10.0178	$[M+H]^+$	$C_{27}H_{30}O_{16}$	611.1624	611.1614	1.67	0.990	3.969 ± 0.087	3.960 ± 0.017	5.418 ± 0.021	5.473 ± 0.029
Quercetin or isomer_2	10.3922	$[M+H]^+$	$C_{15}H_{10}O_7$	303.0508	303.0499	2.96	0.969	1.392 ± 0.016	1.468 ± 0.078	0.385 ± 0.007	0.381 ± 0.008
Isoquercitrin	10.3930	$[M+H]^+$	$C_{21}H_{20}O_{12}$	465.1043	465.1030	2.71	0.986	2.516 ± 0.032	2.330 ± 0.055	1.478 ± 0.023	1.473 ± 0.013
Kaempferol-3-*O*-rutinoside	10.7479	$[M+H]^+$	$C_{27}H_{30}O_{15}$	595.1674	595.1652	3.63	0.991	2.455 ± 0.080	2.453 ± 0.009	3.213 ± 0.033	3.239 ± 0.006
Kaempferol-7-*O*-glucoside	11.1288	$[M+H]^+$	$C_{21}H_{20}O_{11}$	449.1089	449.1078	2.45	0.982	0.938 ± 0.011	0.916 ± 0.025	0.851 ± 0.019	0.848 ± 0.014
Feruloyltyramine	12.0531	$[M+H]^+$	$C_{18}H_{19}NO_4$	314.1397	314.1387	3.23	0.924	0.436 ± 0.011	0.451 ± 0.014	0.381 ± 0.018	0.386 ± 0.002
Tiliroside	13.4122	$[M+H]^+$	$C_{30}H_{26}O_{13}$	595.1449	595.1446	0.58	0.988	0.126 ± 0.011	0.131 ± 0.004	Tr	Tr

Table S3 (continued). Untargeted LC-MS/MS analysis results in positive ion mode.

Compound name	RT (min)	Ion adduct	Molecular formula	Observed *m/z*	Theoretical *m/z*	*m/z* diff (ppm)	Similarity score	Relative peak area (%)			
								HS-6	HS-6A	HS-3	HS-3A
LPC 18:3	16.6737	$[M+H]^+$	$C_{26}H_{48}NO_7P$	518.3258	518.3263	-0.91	0.913	2.528 ± 0.071	2.373 ± 0.120	4.331 ± 0.053	4.297 ± 0.034
LPC 18:2	17.1498	$[M+H]^+$	$C_{26}H_{50}NO_7P$	520.3403	520.3408	-1.03	0.937	1.233 ± 0.055	1.215 ± 0.077	1.921 ± 0.001	1.845 ± 0.003
9,12,15-Octadecatrienoic acid, 3-(hexopyranosyloxy)-2-hydroxypropyl ester, (9*Z*,12*Z*,15*Z*) (monoglycosylmonoacylglycerol (18:3))	17.2574	$[M+Na]^+$	$C_{27}H_{46}O_9$	537.3049	537.3030	3.47	0.886	0.652 ± 0.007	0.620 ± 0.010	1.364 ± 0.044	1.327 ± 0.004
LPE 16:0	17.4495	$[M+H]^+$	$C_{21}H_{44}NO_7P$	454.2941	454.2928	2.80	0.945	0.235 ± 0.011	0.242 ± 0.031	0.416 ± 0.028	0.403 ± 0.011
LPC 16:0	17.4741	$[M+H]^+$	$C_{24}H_{50}NO_7P$	496.3415	496.3398	3.49	0.982	2.791 ± 0.017	2.644 ± 0.133	4.518 ± 0.014	4.287 ± 0.046
LPC 18:1	17.6856	$[M+H]^+$	$C_{26}H_{52}NO_7P$	522.3561	522.3563	-0.39	0.929	0.392 ± 0.016	0.383 ± 0.012	0.698 ± 0.014	0.683 ± 0.015
LPC 18:0	18.3132	$[M+H]^+$	$C_{26}H_{54}NO_7P$	524.3713	524.3711	0.35	0.973	Tr	Tr	0.215 ± 0.025	0.207 ± 0.007
Khivorin	19.6789	$[M+Na]^+$	$C_{32}H_{42}O_{10}$	609.2723	609.2670	8.70	0.886	1.613 ± 0.090	1.768 ± 0.008	5.083 ± 0.136	4.881 ± 0.032
Deoxykhivorin	20.0553	$[M+Na]^+$	$C_{32}H_{42}O_9$	593.2769	593.2732	6.27	0.830	4.496 ± 0.076	5.165 ± 0.129	5.034 ± 0.036	4.919 ± 0.023

Tr represents trace (relative peak area < 0.1%).

Table S4. The abbreviations for the original compound names used in the heatmap of positive ion mode data.

**Abbreviations**	**Original names**
SPD	Spermidine
Agm	Agmatine
Cho	Choline
GABA	γ-Aminobutyric acid
Asn	Asparagine
PCho	Phosphorylcholine
Bet	Betaine
Trig	Trigonelline
Pro	Proline
GPC	*sn*-Glycero-3-phosphocholine
Ade	Adenine
HMP Iso1	3-Hydroxy-2-methyl-4-pyrone or isomer_1
Gua	Guanine
PN	Pyridoxine
Ile	Isoleucine
CumAlc	Cuminyl alcohol
PGlu	Pyroglutamic acid
Tyr	Tyrosine
Ado	Adenosine
Phe	Phenylalanine
HMP Iso2	3-Hydroxy-2-methyl-4-pyrone or isomer_2
IAAc	Indoleacrylic acid
Trp	Tryptophan
MTA	5'-*S*-Methylthioadenosine
N6SA	N^6-Succinyladenosine
RA	Rosmarinic acid
ChlA	Chlorogenic acid
CA	Caffeic acid
THHCA	Tetrahydroharman-3-carboxylic acid
Cou-Glc	(*E*)-3-[4-[(2*S*,3*R*,4*S*,5*S*,6*R*)-3,4,5-trihydroxy-6-(hydroxymethyl)oxan-2-yl]oxyphenyl]prop-2-enoic acid (*p*-coumaric acid-4-*O*-glucoside)
Que-Vic Iso1	2-(3,4-dihydroxyphenyl)-5,7-dihydroxy-3-[(2*S*,3*R*,4*S*,5*S*,6*R*)-3,4,5-trihydroxy-6-[[(2*S*,3*R*,4*S*,5*R*)-3,4,5-trihydroxyoxan-2-yl]oxymethyl]oxan-2-yl]oxychromen-4-one (quercetin-3-*O*-vicianoside) or isomer_1
Cou	Coumarin

Kae-Vic Iso1	5,7-dihydroxy-2-(4-hydroxyphenyl)-3-[(2*S*,3*R*,4*S*,5*S*,6*R*)-3,4,5-trihydroxy-6-[[(2*S*,3*R*,4*S*,5*S*)-3,4,5-trihydroxyoxan-2-yl]oxymethyl]oxan-2-yl]oxychromen-4-one (kaempferol-3-*O*-vicianoside) or isomer_1
Ros	Roseoside
Lol	Loliolide
Que-GlcDiRha Iso1	3-[6-[[(2*R*,3*R*,4*R*,5*S*,6*S*)-3,5-dihydroxy-6-methyl-4-[(2*S*,3*R*,4*R*,5*R*,6*S*)-3,4,5-trihydroxy-6-methyloxan-2-yl]oxyoxan-2-yl]oxymethyl]-3,4,5-trihydroxyoxan-2-yl]oxy-2-(3,4-dihydroxyphenyl)-5,7-dihydroxychromen-4-one (quercetin-3-*O*-glucopyranosyl-dirhamnopyranoside) or isomer_1
Que-Vic Iso2	2-(3,4-dihydroxyphenyl)-5,7-dihydroxy-3-[(2*S*,3*R*,4*S*,5*S*,6*R*)-3,4,5-trihydroxy-6-[[(2*S*,3*R*,4*S*,5*R*)-3,4,5-trihydroxyoxan-2-yl]oxymethyl]oxan-2-yl]oxychromen-4-one (quercetin-3-*O*-vicianoside) or isomer_2
Kae-GlcDiRha Iso1	3-[(2*S*,3*R*,4*S*,5*R*,6*R*)-6-[[(2*R*,3*R*,4*R*,5*S*,6*S*)-3,5-dihydroxy-6-methyl-4-[(2S,3R,4R,5R,6S)-3,4,5-trihydroxy-6-methyloxan-2-yl]oxyoxan-2-yl]oxymethyl]-3,4,5-trihydroxyoxan-2-yl]oxy-5,7-dihydroxy-2-(4-hydroxyphenyl)chromen-4-one (kaempferol-3-*O*-glucopyranosyl-dirhamnopyranoside) or isomer_1
Que Iso1	Quercetin or isomer_1
Rut	Rutin
Que Iso2	Quercetin or isomer_2
IsoQ	Isoquercitrin
Kae-Rut	Kaempferol-3-*O*-rutinoside
Kae-Glc	Kaempferol-7-*O*-glucoside
FTA	Feruloyltyramine
Til	Tiliroside
MGMG 18:3	9,12,15-Octadecatrienoic acid, 3-(hexopyranosyloxy)-2-hydroxypropyl ester, (9*Z*,12*Z*,15*Z*) (monoglycosylmonoacylglycerol (18:3))
LPE 16:0	1-Palmitoyl-*sn*-glycero-3-phosphoethanolamine (lysophosphatidylethanolamine (16:0))
LPC 16:0	1-Hexadecanoyl-*sn*-glycero-3-phosphocholine (lysophosphatidylcholine (16:0))
LPC 18:0	1-Octadecanoyl-*sn*-glycero-3-phosphocholine (lysophosphatidylcholine (18:0))
LPC 18:1	1-Octadecenoyl-*sn*-glycero-3-phosphocholine (lysophosphatidylcholine (18:1))
LPC 18:2	1-Octadecadienoyl-*sn*-glycero-3-phosphocholine (lysophosphatidylcholine (18:2))
LPC 18:3	1-Octadecatrienoyl-*sn*-glycero-3-phosphocholine (lysophosphatidylcholine (18:3))
Khi	Khivorin
DKhi	Deoxykhivorin

Table S5. Untargeted LC-MS/MS analysis results in negative ion mode.

Compound name	RT (min)	Ion adduct	Molecular formula	Observed *m/z*	Theoretical *m/z*	*m/z* diff (ppm)	Similarity score	Relative peak area (%)			
								HS-6	HS-6A	HS-3	HS-3A
Asparagine	0.6977	$[M-H]^-$	$C_4H_8N_2O_3$	131.0463	131.0462	0.54	0.909	3.512 ± 0.009	2.972 ± 0.023	2.023 ± 0.009	2.061 ± 0.016
Aspartic acid	0.7182	$[M-H]^-$	$C_4H_7NO_4$	132.0299	132.0302	-2.32	0.888	0.578 ± 0.005	0.852 ± 0.029	0.567 ± 0.003	0.525 ± 0.004
Glucose	0.7209	$[M-H]^-$	$C_6H_{12}O_6$	179.0565	179.0561	2.42	0.897	1.808 ± 0.019	2.291 ± 0.030	2.079 ± 0.020	2.235 ± 0.007
Sucrose	0.7519	$[M-H]^-$	$C_{12}H_{22}O_{11}$	341.1095	341.1090	1.52	0.951	2.170 ± 0.061	1.287 ± 0.049	1.104 ± 0.021	0.876 ± 0.011
Quinic acid	0.7794	$[M-H]^-$	$C_7H_{12}O_6$	191.0570	191.0561	4.87	0.988	1.565 ± 0.004	1.339 ± 0.028	2.132 ± 0.028	2.790 ± 0.016
Malic acid	0.8102	$[M-H]^-$	$C_4H_6O_5$	133.0145	133.0142	2.33	0.950	3.473 ± 0.014	3.164 ± 0.042	2.687 ± 0.003	2.339 ± 0.005
Citric acid	0.8590	$[M-H]^-$	$C_6H_8O_7$	191.0199	191.0197	1.17	0.993	2.078 ± 0.039	1.865 ± 0.030	1.501 ± 0.015	1.346 ± 0.006
Benzoic acid + 2O	3.0164	$[M-H]^-$	$C_{13}H_{16}O_9$	315.0718	315.0700	5.83	0.889	Tr	0.110 ± 0.003	0.142 ± 0.003	0.152 ± 0.003
Neochlorogenic acid	4.1777	$[M-H]^-$	$C_{16}H_{18}O_9$	353.0886	353.0878	2.22	0.943	10.321 ± 0.013	10.467 ± 0.299	8.504 ± 0.006	8.243 ± 0.076
(1*R*,3*R*,4*S*,5*R*)-1,3,4-trihydroxy-5-[(*E*)-3-(4-hydroxyphenyl)prop-2-enoyl]oxycyclohexane-1-carboxylic acid (5-*p*-coumaroylquinic acid) or isomer_1	5.3105	$[M-H]^-$	$C_{16}H_{18}O_8$	337.0923	337.0929	-1.86	0.836	0.117 ± 0.002	Tr	Tr	Tr
(1*R*,3*R*,4*S*,5*R*)-1,3,4-trihydroxy-5-[(*E*)-3-(4-hydroxyphenyl)prop-2-enoyl]oxycyclohexane-1-carboxylic acid (5-*p*-coumaroylquinic acid) or isomer_2	5.5589	$[M-H]^-$	$C_{16}H_{18}O_8$	337.0925	337.0929	-1.16	0.864	0.350 ± 0.011	0.382 ± 0.008	0.302 ± 0.002	0.304 ± 0.003
(1*S*,3*R*,4*S*,5*R*)-4-{[(2*E*)-3-(3,4-dihydroxyphenyl)prop-2-enoyl]oxy}-1,3,5-trihydroxycyclohexane-1-carboxylic acid (cryptochlorogenic acid) or isomer_1	5.9295	$[M-H]^-$	$C_{16}H_{18}O_9$	353.0882	353.0878	1.05	0.940	2.185 ± 0.007	2.093 ± 0.066	2.302 ± 0.018	2.135 ± 0.085
(1*S*,3*R*,4*S*,5*R*)-4-{[(2*E*)-3-(3,4-dihydroxyphenyl)prop-2-enoyl]oxy}-1,3,5-trihydroxycyclohexane-1-carboxylic acid (cryptochlorogenic acid) or isomer_2	6.2266	$[M-H]^-$	$C_{16}H_{18}O_9$	353.0887	353.0878	2.51	0.943	7.053 ± 0.008	6.263 ± 0.058	7.323 ± 0.095	6.537 ± 0.058

Table S5 (continued). Untargeted LC-MS/MS analysis results in negative ion mode.

Compound name	RT (min)	Ion adduct	Molecular formula	Observed *m/z*	Theoretical *m/z*	*m/z* diff (ppm)	Similarity score	Relative peak area (%) HS-6	HS-6A	HS-3	HS-3A
Chlorogenic acid	6.5430	$[M-H]^-$	$C_{16}H_{18}O_9$	353.0878	353.0878	-0.04	0.982	0.351 ± 0.002	0.343 ± 0.011	0.586 ± 0.005	0.589 ± 0.007
Caffeic acid or isomer_1	6.6321	$[M-H]^-$	$C_9H_8O_4$	179.0346	179.0350	-2.20	0.976	Tr	Tr	0.141 ± 0.001	0.300 ± 0.008
Caffeic acid or isomer_2	6.7026	$[M-H]^-$	$C_9H_8O_4$	179.0347	179.0350	-1.68	0.976	Tr	Tr	0.370 ± 0.002	0.274 ± 0.003
(2*S*,3*S*,4*S*,5*R*,6*R*)-6-(3-benzoyloxy-2-hydroxypropoxy)-3,4,5-trihydroxyoxane-2-carboxylic acid (benzoylglycerol glucuronide)	7.2938	$[M-H]^-$	$C_{16}H_{20}O_{10}$	371.0982	371.0986	-1.14	0.945	0.268 ± 0.000	0.207 ± 0.014	0.389 ± 0.008	0.362 ± 0.003
(2*S*,3*R*,4*S*,5*R*)-2-[(2*R*,3*R*,4*S*,5*S*,6*R*)-4,5-dihydroxy-6-(hydroxymethyl)-2-(2-phenylethoxy)oxan-3-yl]oxyoxane-3,4,5-triol (2-phenylethyl-3-*O*-pentosylglucoside)	7.4039	$[M+FA-H]^-$	$C_{19}H_{28}O_{10}$	461.1654	461.1665	-2.32	0.833	0.130 ± 0.001	0.120 ± 0.007	Tr	Tr
1,3,5-trihydroxy-4-[(*E*)-3-(3-hydroxy-4-methoxyphenyl)prop-2-enoyl]oxycyclohexane-1-carboxylic acid (4-*O*-isoferuloylquinic acid)	8.0667	$[M-H]^-$	$C_{17}H_{20}O_9$	367.1030	367.1040	-2.76	0.970	0.410 ± 0.005	0.406 ± 0.001	0.114 ± 0.001	0.105 ± 0.001
2-(3,4-dihydroxyphenyl)-3-[(2*S*,3*R*,4*S*,5*S*,6*R*)-4,5-dihydroxy-3-[(2*R*,3*R*,4*R*,5*R*,6*S*)-3,4,5-trihydroxy-6-methyloxan-2-yl]oxy-6-[[(2*R*,3*R*,4*R*,5*R*,6*S*)-3,4,5-trihydroxy-6-methyloxan-2-yl]oxymethyl]oxan-2-yl]oxy-5,7-dihydroxychromen-4-one (quercetin-3-*O*-glucopyranosyl-dirhamnopyranoside) or isomer_1	9.3285	$[M-H]^-$	$C_{33}H_{40}O_{20}$	755.2027	755.2040	-1.67	0.999	1.956 ± 0.011	1.687 ± 0.030	1.711 ± 0.012	1.645 ± 0.011
Quercetin-3-*O*-vicianoside	9.7136	$[M-H]^-$	$C_{26}H_{28}O_{16}$	595.1291	595.1305	-2.32	0.972	0.966 ± 0.004	0.536 ± 0.023	0.183 ± 0.007	0.132 ± 0.001

Table S5 (continued). Untargeted LC-MS/MS analysis results in negative ion mode.

Compound name	RT (min)	Ion adduct	Molecular formula	Observed *m/z*	Theoretical *m/z*	*m/z* diff (ppm)	Similarity score	Relative peak area (%)			
								HS-6	HS-6A	HS-3	HS-3A
3-[4,5-dihydroxy-3-(3,4,5-trihydroxy-6-methyloxan-2-yl)oxy-6-[(3,4,5-trihydroxy-6-methyloxan-2-yl)oxymethyl]oxan-2-yl]oxy-5,7-dihydroxy-2-(4-hydroxyphenyl)chromen-4-one (kaempferol-3-*O*-glucopyranosyl-dirhamnopyranoside) or isomer_1	9.8372	[M−H]⁻	$C_{33}H_{40}O_{19}$	739.2079	739.2091	-1.66	0.998	2.038 ± 0.041	1.782 ± 0.013	1.560 ± 0.015	1.490 ± 0.009
Rutin or isomer_1	9.9409	[M−H]⁻	$C_{27}H_{30}O_{16}$	609.1454	609.1461	-1.13	0.989	1.145 ± 0.027	0.889 ± 0.022	1.048 ± 0.018	0.924 ± 0.005
Rutin or isomer_2	10.0289	[M−H]⁻	$C_{27}H_{30}O_{16}$	609.1461	609.1461	0.03	0.993	13.866 ± 0.079	16.056 ± 0.189	17.080 ± 0.039	17.381 ± 0.026
Isoquercitrin	10.4035	[M−H]⁻	$C_{21}H_{20}O_{12}$	463.0889	463.0879	2.10	0.994	8.358 ± 0.073	8.003 ± 0.039	4.540 ± 0.048	4.381 ± 0.027
Kaempferol-3-*O*-rutinoside or isomer_1	10.4351	[M−H]⁻	$C_{27}H_{30}O_{15}$	593.1496	593.1512	-1.80	0.981	0.173 ± 0.012	0.157 ± 0.002	0.185 ± 0.002	0.170 ± 0.004
(2*R*,3*S*,4*S*,5*R*,6*R*)-2-[[(2*R*,3*R*,4*R*)-3,4-dihydroxy-4-(hydroxymethyl)oxolan-2-yl]oxymethyl]-6-oct-1-en-3-yloxyoxane-3,4,5-triol (1-octen-3-yl-6-*O*-apiofuranosyl-glucopyranoside) or isomer_1	10.7164	[M+FA−H]⁻	$C_{19}H_{34}O_{10}$	467.2125	467.2130	-1.11	0.958	0.144 ± 0.004	0.119 ± 0.005	0.136 ± 0.001	0.127 ± 0.001
Kaempferol-3-*O*-rutinoside or isomer_2	10.7584	[M−H]⁻	$C_{27}H_{30}O_{15}$	593.1513	593.1512	0.14	0.997	12.157 ± 0.112	10.804 ± 0.248	13.779 ± 0.044	13.924 ± 0.021
(2*R*,3*S*,4*S*,5*R*,6*R*)-2-[[(2*R*,3*R*,4*R*)-3,4-dihydroxy-4-(hydroxymethyl)oxolan-2-yl]oxymethyl]-6-oct-1-en-3-yloxyoxane-3,4,5-triol (1-octen-3-yl-6-*O*-apiofuranosyl-glucopyranoside) or isomer_2	11.0064	[M+FA−H]⁻	$C_{19}H_{34}O_{10}$	467.2124	467.2130	-1.25	0.940	0.212 ± 0.008	0.178 ± 0.007	0.148 ± 0.002	0.134 ± 0.002

Table S5 (continued). Untargeted LC-MS/MS analysis results in negative ion mode.

Compound name	RT (min)	Ion adduct	Molecular formula	Observed *m/z*	Theoretical *m/z*	*m/z* diff (ppm)	Similarity score	Relative peak area (%)			
								HS-6	HS-6A	HS-3	HS-3A
Kaempferol 7-*O*-glucoside	11.1407	[M−H]⁻	$C_{21}H_{20}O_{11}$	447.0937	447.0933	0.95	0.986	3.256 ± 0.009	2.870 ± 0.028	1.608 ± 0.009	1.504 ± 0.017
(10*E*,15*Z*)-9,12,13-trihydroxyoctadeca-10,15-dienoic acid or isomer_1	13.1735	[M−H]⁻	$C_{18}H_{32}O_5$	327.2175	327.2178	-0.88	0.918	Tr	Tr	0.495 ± 0.007	0.353 ± 0.008
α-Linolenic acid derivative or isomer_1	13.3317	[M−H]⁻	$C_{18}H_{32}O_5$	327.2175	327.2163	3.80	0.937	Tr	Tr	0.999 ± 0.010	0.759 ± 0.010
Tiliroside	13.4140	[M−H]⁻	$C_{30}H_{26}O_{13}$	593.1289	593.1301	-2.04	0.999	0.626 ± 0.003	0.563 ± 0.009	0.235 ± 0.002	0.230 ± 0.001
α-Linolenic acid derivative or isomer_2	13.4163	[M−H]⁻	$C_{18}H_{32}O_5$	327.2174	327.2163	3.51	0.912	Tr	Tr	0.868 ± 0.004	0.715 ± 0.004
α-Linolenic acid derivative or isomer_3	13.5519	[M−H]⁻	$C_{18}H_{32}O_5$	327.2172	327.2163	2.69	0.896	Tr	Tr	0.218 ± 0.005	0.176 ± 0.004
Quercetin	13.6072	[M−H]⁻	$C_{15}H_{10}O_7$	301.0363	301.0354	2.83	0.982	4.885 ± 0.075	6.713 ± 0.178	4.096 ± 0.024	5.969 ± 0.167
(*Z*)-5,8,11-trihydroxyoctadec-9-enoic acid	13.7923	[M−H]⁻	$C_{18}H_{34}O_5$	329.2327	329.2334	-2.13	0.960	Tr	Tr	0.176 ± 0.001	0.121 ± 0.001
Kaempferol	15.0524	[M−H]⁻	$C_{15}H_{10}O_6$	285.0413	285.0405	2.68	0.994	1.441 ± 0.035	3.220 ± 0.148	1.587 ± 0.005	2.524 ± 0.026
Glc-Glc-octadecatrienoyl-*sn*-glycerol (diglucosylmonoacylglycerol (18:3)) or isomer_1	16.3780	[M+HCOO]⁻	$C_{33}H_{56}O_{14}$	721.3637	721.3652	-2.08	0.838	0.542 ± 0.011	0.489 ± 0.012	1.104 ± 0.005	0.999 ± 0.013
Glc-Glc-octadecatrienoyl-*sn*-glycerol (diglucosylmonoacylglycerol (18:3)) or isomer_2	16.5827	[M+HCOO]⁻	$C_{33}H_{56}O_{14}$	721.3648	721.3652	-0.55	0.840	6.165 ± 0.092	6.519 ± 0.052	10.118 ± 0.006	9.454 ± 0.082
LPE 16:0	17.4521	[M−H]⁻	$C_{21}H_{44}NO_7P$	452.2786	452.2766	4.40	0.889	1.336 ± 0.007	1.188 ± 0.077	1.754 ± 0.008	1.691 ± 0.046
LPC 16:0	17.4817	[M+HCOO]⁻	$C_{24}H_{50}NO_7P$	540.3311	540.3307	0.69	0.992	3.668 ± 0.016	3.498 ± 0.020	3.949 ± 0.006	3.823 ± 0.055

Tr represents trace (relative peak area < 0.1%).

Table S6. The abbreviations for the original compound names used in the heatmap of negative ion mode data.

**Abbreviations**	**Original names**
Asn	Asparagine
Asp	Aspartic acid
Glc	Glucose
Suc	Sucrose
QA	Quinic acid
Mal	Malic acid
CAcid	Citric acid
BzA-2O	Benzoic acid + 2O
NeoA	Neochlorogenic acid
CouQA Iso1	(1*R*,3*R*,4*S*,5*R*)-1,3,4-trihydroxy-5-[(E)-3-(4-hydroxyphenyl)prop-2-enoyl]oxycyclohexane-1-carboxylic acid (5-*p*-coumaroylquinic acid) or isomer_1
CouQA Iso2	(1*R*,3*R*,4*S*,5*R*)-1,3,4-trihydroxy-5-[(E)-3-(4-hydroxyphenyl)prop-2-enoyl]oxycyclohexane-1-carboxylic acid (5-*p*-coumaroylquinic acid) or isomer_2
CryA Iso1	(1*S*,3*R*,4*S*,5*R*)-4-{[(2E)-3-(3,4-dihydroxyphenyl)prop-2-enoyl]oxy}-1,3,5-trihydroxycyclohexane-1-carboxylic acid (cryptochlorogenic acid) or isomer_1
CryA Iso2	(1*S*,3*R*,4*S*,5*R*)-4-{[(2E)-3-(3,4-dihydroxyphenyl)prop-2-enoyl]oxy}-1,3,5-trihydroxycyclohexane-1-carboxylic acid (cryptochlorogenic acid) or isomer_2
ChlA	Chlorogenic acid
CA Iso1	Caffeic acid or isomer_1
CA Iso2	Caffeic acid or isomer_2
BzGlyGlcA	(2*S*,3*S*,4*S*,5*R*,6*R*)-6-(3-benzoyloxy-2-hydroxypropoxy)-3,4,5-trihydroxyoxane-2-carboxylic acid (benzoylglycerol glucuronide)
PE-PenGlc	(2*S*,3*R*,4*S*,5*R*)-2-[(2*R*,3*R*,4*S*,5*S*,6*R*)-4,5-dihydroxy-6-(hydroxymethyl)-2-(2-phenylethoxy)oxan-3-yl]oxyoxane-3,4,5-triol (2-phenylethyl-3-*O*-pentosylglucoside)
IFQA	1,3,5-trihydroxy-4-[(*E*)-3-(3-hydroxy-4-methoxyphenyl)prop-2-enoyl]oxycyclohexane-1-carboxylic acid (4-*O*-isoferuloylquinic acid)
Que-GlcDiRha Iso1	2-(3,4-dihydroxyphenyl)-3-[(2*S*,3*R*,4*S*,5*S*,6*R*)-4,5-dihydroxy-3-[(2*R*,3*R*,4*R*,5*R*,6*S*)-3,4,5-trihydroxy-6-methyloxan-2-yl]oxy-6-[[(2*R*,3*R*,4*R*,5*R*,6*S*)-3,4,5-trihydroxy-6-methyloxan-2-yl]oxymethyl]oxan-2-yl]oxy-5,7-dihydroxychromen-4-one (quercetin-3-*O*-glucopyranosyl-dirhamnopyranoside) or isomer_1
Que-Vic Iso1	Quercetin-3-*O*-vicianoside or isomer_1

Kae-GlcDiRha Iso1	3-[4,5-dihydroxy-3-(3,4,5-trihydroxy-6-methyloxan-2-yl)oxy-6-[(3,4,5-trihydroxy-6-methyloxan-2-yl)oxymethyl]oxan-2-yl]oxy-5,7-dihydroxy-2-(4-hydroxyphenyl)chromen-4-one (kaempferol-3-*O*-glucopyranosyl-dirhamnopyranoside) or isomer_1
Rut Iso1	Rutin or isomer_1
Rut Iso2	Rutin or isomer_2
IsoQ	Isoquercitrin
Kae-Rut Iso1	Kaempferol-3-*O*-rutinoside or isomer_1
Oct-ApiGlc Iso1	(2*R*,3*S*,4*S*,5*R*,6*R*)-2-[[(2*R*,3*R*,4*R*)-3,4-dihydroxy-4-(hydroxymethyl)oxolan-2-yl]oxymethyl]-6-oct-1-en-3-yloxyoxane-3,4,5-triol (1-octen-3-yl-6-*O*-apiofuranosyl-glucopyranoside) or isomer_1
Kae-Rut Iso2	Kaempferol-3-*O*-rutinoside or isomer_2
Oct-ApiGlc Iso2	(2*R*,3*S*,4*S*,5*R*,6*R*)-2-[[(2*R*,3*R*,4*R*)-3,4-dihydroxy-4-(hydroxymethyl)oxolan-2-yl]oxymethyl]-6-oct-1-en-3-yloxyoxane-3,4,5-triol (1-octen-3-yl-6-*O*-apiofuranosyl-glucopyranoside) or isomer_2
Kae-Glc	Kaempferol-7-*O*-glucoside
THODA Iso1	(10*E*,15*Z*)-9,12,13-trihydroxyoctadeca-10,15-dienoic acid or isomer_1
LNA Iso1	α-Linolenic acid derivative or isomer_1
Til	Tiliroside
LNA Iso2	α-Linolenic acid derivative or isomer_2
LNA Iso3	α-Linolenic acid derivative or isomer_3
Que	Quercetin
THOA	(*Z*)-5,8,11-trihydroxyoctadec-9-enoic acid
Kae	Kaempferol
DGMG 18:3 Iso1	Glc-Glc-octadecatrienoyl-*sn*-glycerol (diglucosylmonoacylglycerol (18:3)) or isomer_1
DGMG 18:3 Iso2	Glc-Glc-octadecatrienoyl-*sn*-glycerol (diglucosylmonoacylglycerol (18:3)) or isomer_2
LPC 16:0	1-Hexadecanoyl-*sn*-glycero-3-phosphocholine (lysophosphatidylcholine (16:0))
LPE 16:0	1-Palmitoyl-*sn*-glycero-3-phosphoethanolamine (lysophosphatidylethanolamine (16:0))
